# Assessing the influence of autumnal temperature fluctuations on cold hardiness in different grapevine cultivars: variations across vine age and bud positions

**DOI:** 10.3389/fpls.2024.1379328

**Published:** 2024-05-17

**Authors:** Ozkan Kaya, Hava Delavar, Avery Shikanai, Collin Auwarter, Harlene Hatterman-Valenti

**Affiliations:** ^1^ Erzincan Horticultural Research Institute, Republic of Türkiye Ministry of Agriculture and Forestry, Erzincan, Türkiye; ^2^ Department of Plant Sciences, North Dakota State University, Fargo, ND, United States

**Keywords:** autumn temperature fluctuations, bud position, DTA, grapevine, LT50 thresholds, *Vitis* spp

## Abstract

The dynamic fluctuations in autumn temperatures, particularly the marked diurnal variations and the subsequent precipitous drops are key and a pivotal role in viticulture, as they critically influence the acclimation process of grapevines to cold, thereby directly impacting their survival and productivity in cold-climate regions. In this comprehensive study, we investigated the cold hardiness of four grapevine cultivars: ‘Itasca’, ‘Frontenac’, ‘La Crescent’, and ‘Marquette’, focusing on how these cultivars and their individual buds (1^st^, 2^nd^, 3^rd^, 4^th^, 5^th^, 6^th^, 7^th^, 8^th^, and 9^th^) respond to fluctuating weather and low temperatures typical of autumn [-1.1°C (30°F) -9.4°C (15°F) and -17.8°C (0°F)]. Our results illuminated the striking variability in cold hardiness that was manifest not only among the different cultivars but also within individual buds on the same vine, underscoring the critical influence of bud position on a vine for cold hardiness. ‘Frontenac’ showed greater cold hardiness at critical temperatures at which 10%, and 50% of the dormant buds were lethally affected by cold (LT_10_ and LT_50_) compared to ‘Itasca’ and ‘La Crescent’, with ‘Marquette’ exhibiting intermediate values. However, in cultivars such as ‘Itasca’ and ‘Marquette’, certain buds demonstrated a pronounced hardiness when faced with colder temperatures, while others exhibited a heightened sensitivity, thereby revealing a nuanced interplay between bud position and a vine’s ability to withstand cold stress. Our study revealed a notable divergence from traditional viticulture understanding; apical buds demonstrated greater cold hardiness than basal buds and opened new paths for research into grapevine physiology. Our results also indicated a significant trend wherein older vines across all studied cultivars displayed enhanced cold hardiness, particularly pronounced at the critical LT_50_ and the critical temperature at which 90% of the dormant buds were lethally affected by cold (LT_90_) thresholds, in comparison to younger vines. Moreover, our findings shed light on the impact of autumn’s diurnal temperature variations and the subsequent drop in temperatures on vine cold hardiness, thus highlighted the complex interplay between environmental temperature dynamics and dormant bud hardiness. In conclusion, our study showed that the cold damage observed in grapevines in North Dakota was not a result of extreme temperature fluctuations in the fall. This was confirmed by testing the vines after they had reached various threshold temperatures through differential thermal analysis (DTA) and optical differential nucleation and expansion analysis (ODNEAL) methodologies, particularly before the onset of severe pre-winter cold conditions. These comprehensive findings highlighted the complexity of the vine’s response to climatic conditions and viticultural management, pointing to the need for specific strategies in vineyard management and cultivar selection to optimize bud hardiness and productivity in the face of various environmental challenges, especially in cold climate viticulture.

## Introduction

Cold hardiness in grapevines is a multifaceted phenomenon that entails an intricate interplay of myriad complex metabolic and physiological processes, rendering the life cycle of *Vitis* species profoundly susceptible to the changes of seasonal transitions and temperature fluctuations (Kaya and Kose, 2017; Kaya, 2020; Kaya and Kose, 2020; Kose et al., 2024). Changes in temperature and photoperiod depending on the season are the most important factor in activating different physiological mechanisms in grapevines (Fennell, 2004; Rende et al., 2018). In particular, the decrease in day lengths and temperatures serve as signaling mechanisms that initiate these changes and therefore activate histological, biochemical and gene expression mechanisms in these vines, causing an increase in hardiness to low temperatures (Jones, 2005; Jun et al., 2021). These adaptations are directly linked to the vine’s cycle of acclimation, hardening, and de-acclimation stages (Köycü et al., 2017; Keller, 2020). Cold hardiness in grapevines is considered as an integrated process, with these periods overlapping and succeeding each other, making it challenging to define and distinguish their boundaries (Kaya and Kose, 2020). As occurs in many woody species, grapevine acclimation occurs in two stages (Grant, 2012). The first stage begins with growth cessation due to shortened day lengths, decreased temperatures, and limited water consumption (Levitt, 1980; Sakai and Larcher, 1987). During this phase, the plant initiates morphological and physiological changes to tolerate lower temperatures, including growth reduction, induction of dormancy in winter buds, increased partial cold hardiness, and biochemical changes to protect cellular components against environmental low-temperature stresses (Levitt, 1980; Sakai and Larcher, 1987; Levitt, 2012). The decrease in day length around the third week of August in the temperate zone of the Northern Hemisphere, which influences grapevine acclimation, significantly affects biochemical substance changes (Ahmedullah, 1985). Moreover, shorter day lengths enhance periderm development, accelerating the acclimation stage, triggering earlier dormancy, and increasing hardiness to lower temperatures (Fennell and Mathiason, 2002). However, the impact of shortened day lengths on cold hardiness is not as pronounced as the decreasing air temperatures. Although shortening days contribute to the onset of dormancy in buds and shoots (Mills et al., 2006), a decline in air temperature is essential for the plant to develop cold hardiness and transition to the hardening phase (Londo and Kovaleski, 2019; Yilmaz and Fennel, 2021).

The second stage of acclimation, independent of day length shortening, coincides with the fall when air temperatures decrease. This stage is primarily driven by falling air temperatures, without which the plant cannot achieve hardiness and maximum hardiness to winter cold (Jones, 2005). During the second stage, leaf abscission, significant changes in internal metabolites, and maturation in shoots and winter buds are reported (Hamman et al., 1990). The increase in tolerance to low temperatures generally coincides with the first freezing cold events along with substantial changes at the molecular, cellular, and organ levels, leading to reduced water content in tissues and organs (Wolpert and Howell, 1985a, Wolpert and Howell, 1985b; Grant, 2012). Additionally, acclimation leads to decreased cell wall pore sizes and apoplastic permeability in tissues (Rajashekar and Lafta, 1996). Conversely, during the winter months, temperatures that drop below 0°C trigger the transition from the acclimation to the hardening phase in grapevines (Fennell, 2004; Zabadal et al., 2007). During these months, temperatures approach their minimum, and grapevines frequently encounter temperature fluctuations (Zabadal et al., 2007; Grant, 2012). To survive under these conditions, grapevines develop various strategies, generally involving interactions of morphological, physiological, and biochemical substances (Zhang et al., 2012; Rende et al., 2018). It has indeed been noted that a drop in air temperature to -10°C at the beginning of winter increases starch accumulation in grapevine tissues and organs (Eifert et al., 1961). Furthermore, the hardening phase is characterized by a 50–80% reduction in tissue water content (Keller, 2020), as there is a direct relationship between reduced water content and increased cold hardiness (Hamman and Dami, 2000). Subsequently, further temperature decreases break down the starch into sugars, enhancing the plant’s frost tolerance or cold hardiness (Eifert et al., 1961; Koussa et al., 1994). In the light of this information, it shows clearly that it is quite difficult to distinguish the acclimation, hardening and de-acclimation stages in grapevines and to define their boundaries. During these processes, significant changes occur in both primary and secondary endogenous metabolites. Although climatic events significantly affect these metabolite changes, the acclimation, hardening, and de-acclimation stages are significantly affected by freezing events caused by autumn, winter and spring weather conditions. However, there is a notable lack of information in the literature on how off-season climate fluctuations affect the cold hardiness of tissues and organs in different grape species and varieties. Previous research on the ‘Karaerik’ grape cultivar indicated that during the periods of acclimation, hardening, and de-acclimation, the instantaneous temperature changes experienced by winter buds significantly affected their cold hardiness (Kaya and Kose, 2020). This finding indicates the complexity of grapevine response to temperature changes and highlights the need for further research to understand the impact of climatic variations on grapevine hardiness across different seasons.

In this context, it is clear that new studies are necessary to develop more resistant vine varieties and improve viticulture practices to mitigate the effects of climate change and unpredictable weather conditions. Kaya and Kose (2020) reported that instantaneous temperature fluctuations in tissues between the time of sample collection and the beginning of testing can alter lethal temperatures in dormant buds. Based on the current study, it appears that the change in time and temperature between the temperature at the time of sample collection and the initial temperature for testing may change the death threshold in dormant buds. It can, therefore, be assumed that naturally occurring inconsistent, fluctuating, or sudden temperature changes in autumn can cause a significant impact on the cold tolerance of grapevine buds and organs. Building upon this hypothesis, the impact of instantaneous temperature changes in the natural environment during the dormant period on the threshold values of different grapevine species and varieties to low temperatures can be elucidated by monitoring these sudden temperature fluctuations. Based on this hypothesis, we sought response to the following questions with the current research: (i) How do ‘Itasca’, ‘Frontenac’, ‘La Crescent’, and ‘Marquette’ grape cultivars respond to fluctuating low temperatures during the fall? (ii) Does the age of the vine (specifically comparing one and eight-year-old vines) affect its cold hardiness? (iii) Are there observable differences in cold hardiness between these grape cultivars depending on their growing location? (iv) What are the differences in cold hardiness among the first nine buds on the cane?

## Materials and methods

### Plant materials

**Figure 1:**
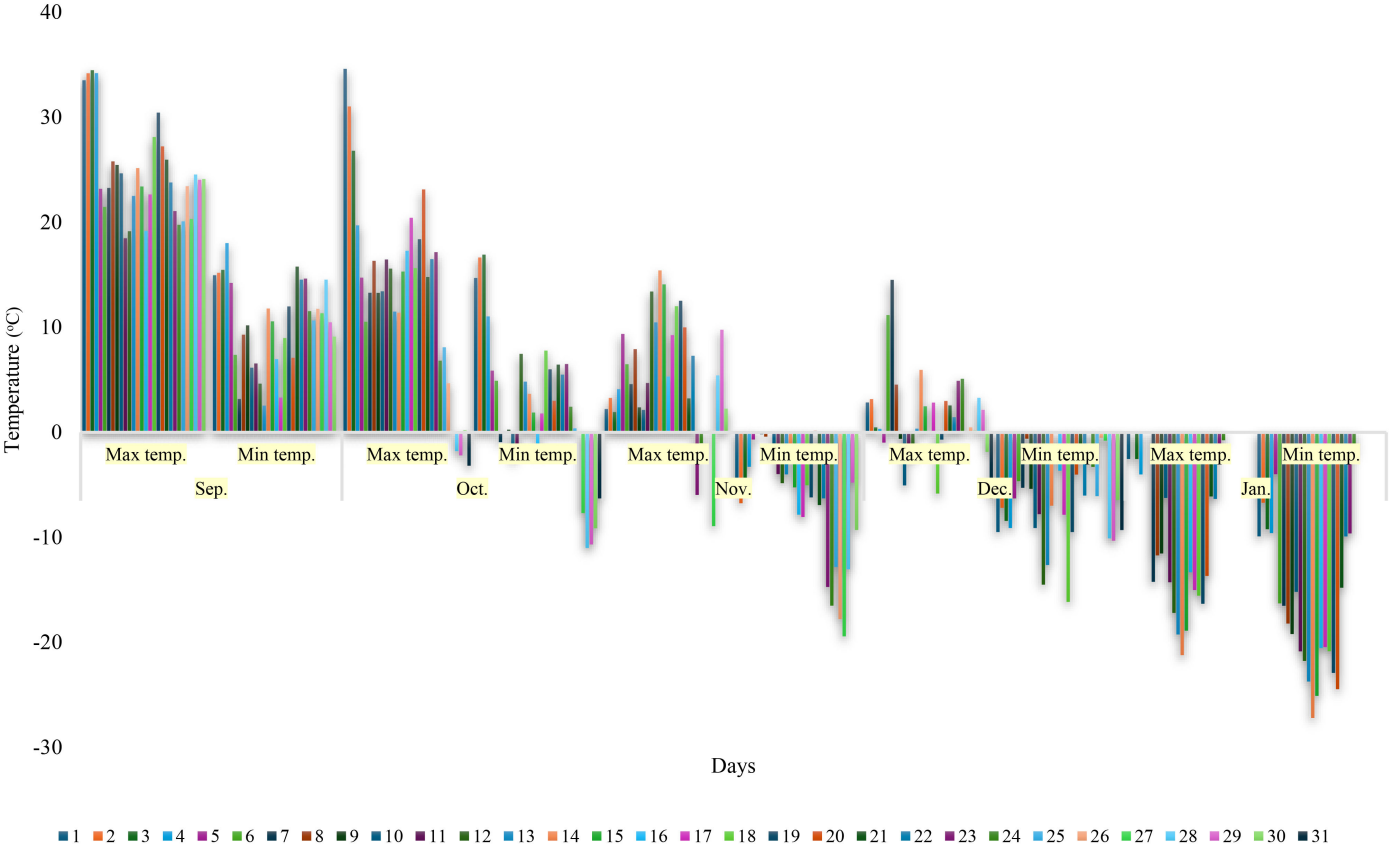
Daily maximum and minimum temperature data over a five-month period (September to January).

The investigation focused on the assessment of dormant buds located at the 1^st^ (most basal) through 9^th^ nodes of one-year-old canes (buds-1, buds-2, buds-3, buds-4, buds-5, buds-6, buds-7, buds-8, and buds-9), which were sourced from vineyards aged eight years and one year, respectively, located at the North Dakota State University Horticulture Research Farm (NDSU-HRF-located near Absaraka) (46° 98’ 94.92” N and -97° 35’ 58.07 “ W) and the NDSU Campus Agricultural Research Center in Fargo, USA (46° 89’ 21.76” N and -96° 81’ 39.57” W). Given the established knowledge that lignification in grapevine canes initiates at the basal bud and progresses towards the apical bud, the study meticulously designed its methodology to account for this gradient in tissue maturity and hardiness (Fennell, 2004). Therefore, to determine if the bud mortality observed in spring was due to the fluctuating frosts experienced in autumn, the buds underwent individual analyses. In the study, four grape cultivars were utilized, each with distinct genetic backgrounds and crossing IDs. Frontenac, identified by the crossing ID and synonym MN 1047, is a pedigree resulting from the cross between ‘Landot 4511’ and the University of Minnesota 89, specifically ‘Landot 4511’ crossed with Riparia 89. La Crescent, another cultivar in the study, is known by the synonym MN 1166 and has a pedigree of ‘St. Pepin’ crossed with E.S. 6–8-25, which is a combination of *V. riparia* and ‘Muscat Hamburg’. This cultivar is an interspecific hybrid comprising 45% *V. vinifera*, 28% *V. riparia*, and smaller proportions of *V. rupestris, V. labrusca*, and *V. aestivalis*. Marquette, referred to by the synonym MN 1211, originates from a complex hybrid involving MN 1094 (a complex hybrid of *V. riparia, V. vinifera*, and other *Vitis* species) and Ravat 262, which is an offspring of ‘Pinot noir’. This makes Marquette an interspecific hybrid that includes *V. riparia, V. vinifera*, and other *Vitis* species. Lastly, the study includes Itasca grapes, identified by the synonym MN1825, which are a product of crossing Frontenac gris with MN1234, the latter being a cross between MN1095 and Seyval. In the eight-year-old vineyard, vines were allocated with a spacing of 2.5 m × 3 m (vine × row), adhering to regular fertilization and irrigation schedules, with each vine bearing 8 to 9 spur-pruned vines. In our study, regular fertilization schedules are meticulously designed around the vine’s developmental cycle, soil nutrient profiles, and the characteristics of the chosen fertilizers, with the objective of administering a balanced nutrient mix at pivotal points. Our irrigation approach is tailored to local climatic patterns, current weather conditions, soil moisture content, and the vine’s stage-specific hydration needs. However, since the annual rainfall was generally 508 mm higher, irrigation was not carried out for the vineyard. Since the organic matter and mineral content of the vineyard soil is high, 19–19-19 NPK granular fertilizer was applied to the vineyard every two years at a rate of 5.6 kg/da. In the spring, this fertilizer was buried 0.25 meters deep on two sides of the grapevine row to prevent interference between the plants and positioned 0.30 meters from the grapevine’s central trunk. In our vineyard for the eight-year-old vines, grape cultivation is conducted using the double cordon training system, which allows for robust vine growth and optimal berry production. Conversely, the one-year-old vines were cultivated in 26 L pots at a closer spacing of 1.5 m × 1.5 m (row × vine), also receiving consistent fertilization and irrigation. One-year-old vines were trained in a single trunk system by tying the vine’s trunk to a bamboo stake for upright growth. Fertilization schedules are meticulously designed around the vine’s developmental cycle, soil nutrient profiles, and the characteristics of the chosen fertilizers, with the objective of administering a balanced nutrient mix at pivotal points, during bud emergence. Our irrigation approach is tailored to local climatic patterns, current weather conditions, soil moisture content in pots, and the vine’s stage-specific hydration needs. For each potted plant, a 3.79 L/h emitter was scheduled to deliver drip irrigation to plants every morning for 30 minutes; and additional irrigation was provided as needed. Approximately 100g of Multicote 4 (14–14-16) (Haifa North America, Savannah GA) was incorporated into the top 10 cm of the growing media on July 17, 2023. Both eight- and one-year old vines were cultivated ungrafted. The experimental design was structured to include three replicates, each comprising 9 vines. The research incorporated the monitoring of climatic data, specifically critical temperatures of -1.1°C, -9.4°C and -17.8°C, crucial for evaluating the immediate impacts of temperature fluctuations on the vines within the region. This climatic data was collected through the North Dakota Agricultural Weather Network (NDAWN) at https://ndawn.ndsu.nodak.edu/weather-data-daily.html (**Figure 1**). Sampling was conducted on three distinct dates, corresponding with the onset of critical temperature thresholds: October 8, 2023, for -1.1°C; November 24, 2023, for -9.4°C; and January 9, 2024, for -17.8°C. The final sampling was strategically timed to precede the winter’s minimum temperatures, providing insights into potential cold damage following the vines’ exposure to fluctuating cold conditions during the fall. This approach facilitated the determination of low-temperature thresholds for the grape cultivars just before the mid-winter minimum temperatures. The selection criteria for canes included health (indicated by dark brown periderm sections), exposure to sunlight, medium size (6–8 mm internode diameter), and the presence of developed periderm on 10 or more internodes. In the study, hedged pruning was not performed on either the eight-year-old or one-year-old shoots. The shoots on each vine were generally divided into 14–15 nodes. However, the study tested the buds found in the first nine nodes. The shoot length in eight-year-old vines was approximately 140–150 cm, while in one-year-old vines, it ranged from 90–100 cm. No yield was obtained from the one-year-old vines within the scope of this study; however, the eight-year-old vines did produce yield. Differential Thermal Analysis (DTA) was performed on the samples after leaf fall. Canes were randomly collected from all vines, with each sampling time yielding a total of 110–120 canes. The collection process was conducted an hour before sunrise to minimize environmental variations. The canes were then stored in polyethylene bags to prevent water loss and desiccation according to the methodology proposed by Kovács et al. (2003). Upon arrival at the laboratory, the samples were divided into two groups: the first group was subjected to DTA, while the second group was analyzed using optical differential nucleation and expansion analysis (ODNEAL).

### Evaluation of cold hardiness of the buds with DTA

**Figure 2:**
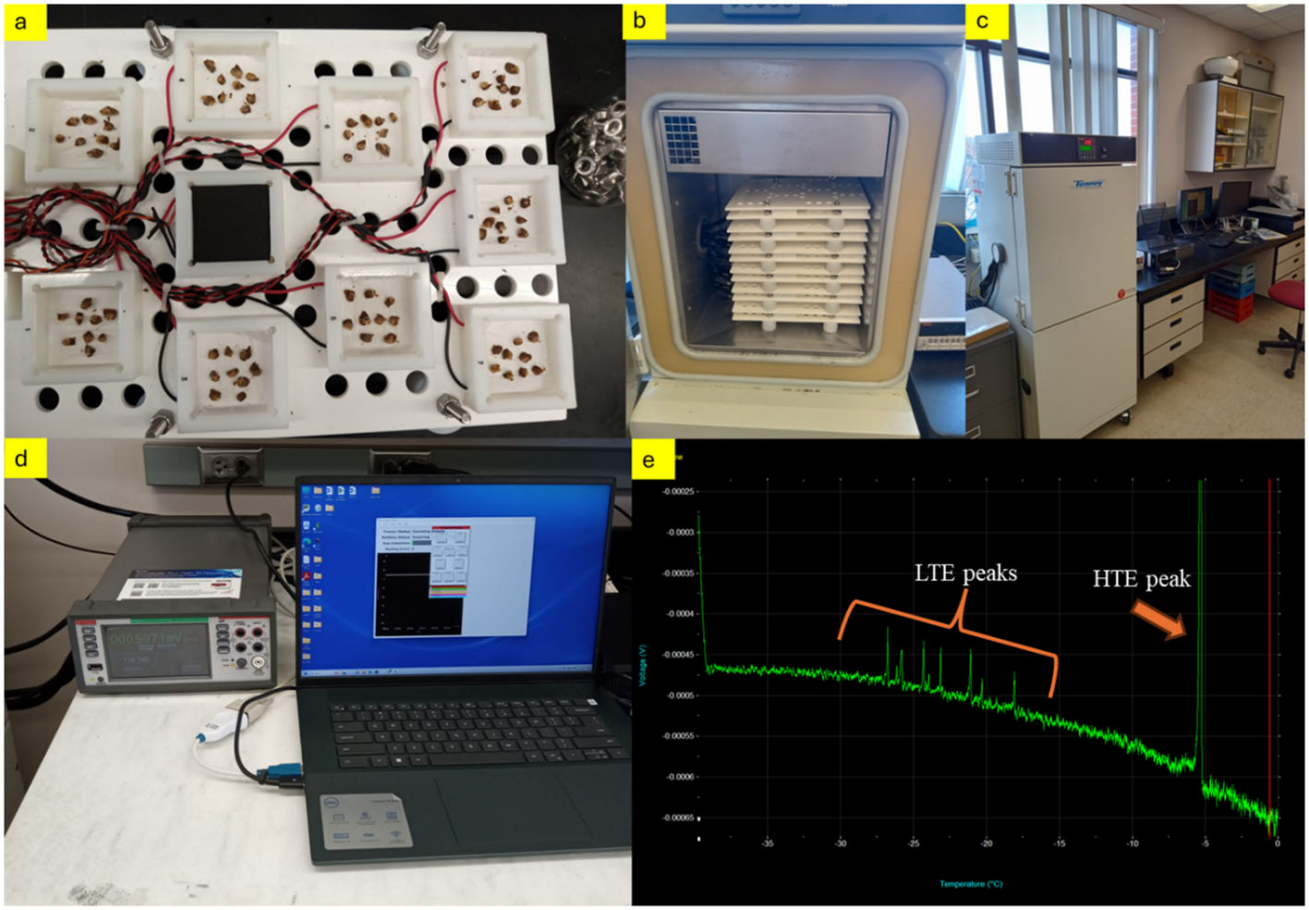
The process involved first placing the buds on the thermoelectric module trays **(A)**, followed by positioning these trays onto the thermal profile system (TPS) device **(B)**. Next, the DTA system was operated **(C)**, during which data was continuously collected and saved to a computer **(D)**. The analysis concluded with identifying two critical thermal events in the buds: the high-temperature exotherm, indicative of nonlethal extracellular freezing, and the low-temperature exotherm, signaling lethal intracellular freezing **(E)** (*Photos by Ozkan Kaya*).

To evaluate the effect of bud position on cold hardiness, harvested canes were separated and randomly divided into three separate groups for each grape cultivar. To determine the different levels of cold hardiness extending from basal to apical regions throughout the canes, a uniform number of dormant buds from identified nodes were selected for research. The principal assessment of bud cold hardiness, inclusive of low-temperature exotherms (LTE), was executed employing DTA, based on the method of Kaya and Kose (2020). Buds ranging from the 1^st^ (most basal) to the 9^th^ node (from buds-1 to buds-9) were precisely excised, with care taken to preserve approximately 2 mm of surrounding tissue intact. Subsequently, these buds were positioned on a thermo-electric module (TEM) within a Tenney Junior Environmental Test Chamber (model T2C-A -F4T, Thermal Product Solutions, New Columbia, PA), which was outfitted with a temperature controller (Test Equity LLC 6100 Condor Drive Moorpark, CA 93021). In the experimental setup, buds from the 1^st^ to the 9^th^ nodes were systematically placed in corresponding wells on TEM trays, numbered from 1^st^ to 9^th^. The study was designed to include three replicates, with each replicate comprising nine buds, thereby necessitating the analysis of 27 buds from each node for every grape cultivar at each sampling interval. The freezer chamber could accommodate up to six trays, each tray holding nine modules, thus allowing for a maximum loading of 54 TEMs per cycle (equating to 486 buds). The freezer chamber’s protocol commenced with a stabilization phase at 4°C for 1 hour, followed by a controlled decrease in temperature from 4°C to -44°C at a decrement rate of 4°C per hour (**Figure 2**). For each DTA assessment, the electrical voltage output from the TEMs, measured in millivolts (mV), was recorded on a computer. This analysis facilitated the calculation of the critical temperatures at which 10%, 50%, the average, and 90% of the dormant buds were lethally affected by cold, denoted as LT_10_, LT_50_, (mLT), and LT_90_, respectively. These critical temperature thresholds were established following the methodology outlined by Kaya and Kose (2020), providing a quantifiable measure of the cold hardiness of the grapevine buds under study.

### Bud browning assay

**Figure 3:**
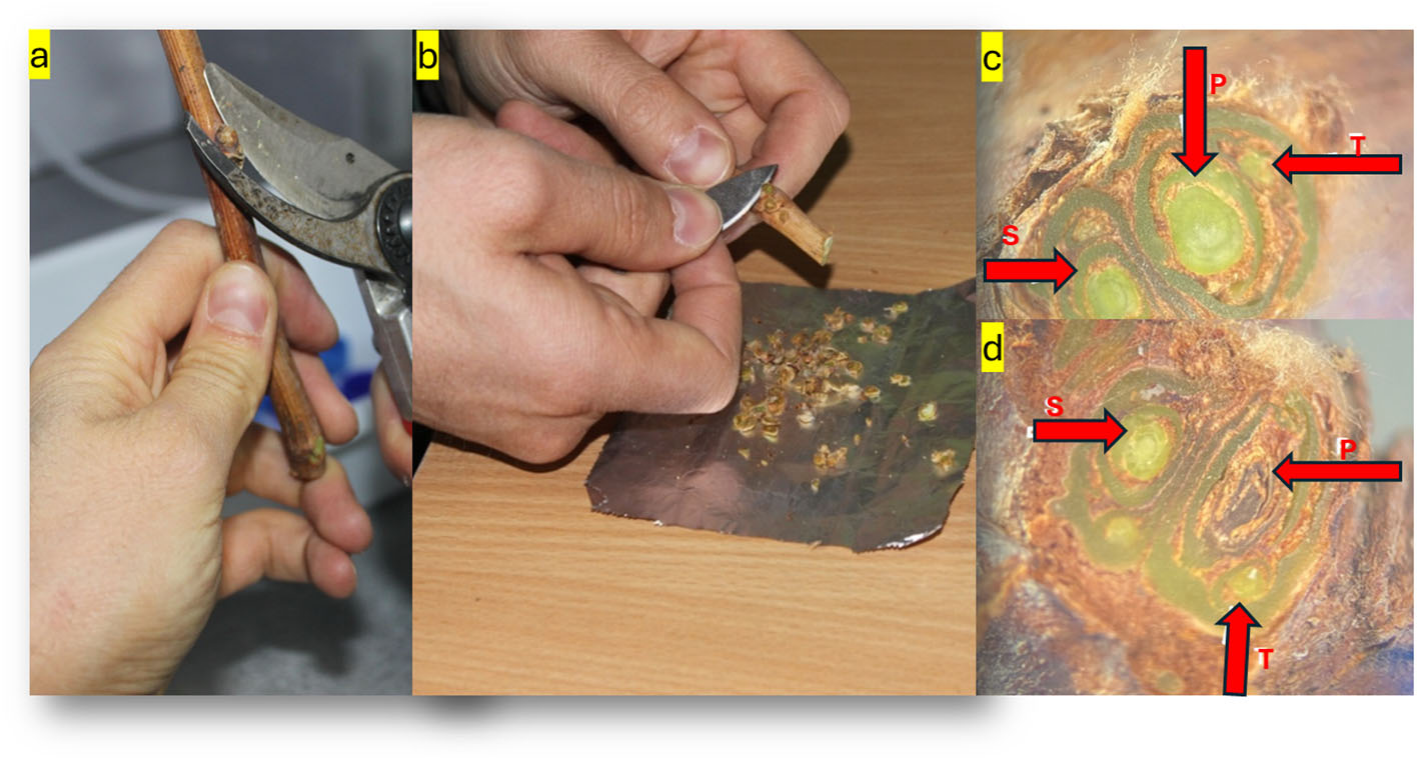
Preparation of buds according to the ODNEAL method **(A)**. Determination of the viability rate in the buds **(B)**. Frost damage in primary, secondary, and tertiary buds **(C)** - all three alive). Scenario where the primary bud is dead while the other two remain alive **(D)** (*Photos by Ozkan Kaya*).

Canes collected following exposure to temperatures of -1.1°C, -9.4°C; and -17.8°C, in vineyard conditions, were subsequently maintained at room temperature for a duration of 48 hours. After this period of acclimatization, diagnostic sectioning was performed on the dormant buds using a single-edged razor blade, facilitating both longitudinal and cross-sectional analyses. Buds ranging from the 1^st^ (most basal) to the 9^th^ node (from buds-1 to buds-9) were precisely excised (**Figure 3**). At each sampling interval, 27 buds from each node were examined for each grape cultivar. This meticulous process aimed to reveal any internal freeze damage, which is typically indicated by a noticeable change in tissue coloration, particularly browning, indicative of cellular injury or death. Buds exhibiting such brown tissue were assessed as non-viable and hence categorized as dead. In contrast, buds retaining vibrant green tissue were evaluated as living and thus deemed viable. These assessments were conducted with the aid of a stereomicroscope, focusing exclusively on the primary buds to ascertain the presence of discoloration, aligning with the methodology delineated by (Stergios and Howell, 1977). The incidence of freeze-induced injury across the grape cultivars was quantified by calculating the proportion of damaged buds. This calculation was based on the observed mortality rate among the buds, adhering to the evaluative criteria established by Odneal (1984).

### Statistical analysis

All LTE values obtained from the DTA were presented as mean ± standard error (SE), derived from three independent replicates to ensure the reliability and reproducibility of the data. In the context of evaluating the hardiness of grapevine buds to cold stress, the following variables were defined to model the adjustments in cold hardiness levels due to varying degrees of temperature-induced stress:

*X_a_* was designated to represent the baseline physiological metric of the grapevine buds, (e.g., maximum cold hardiness level or lowest LTE value),
*X_b_* quantified the magnitude of change in this baseline metric resulting from exposure to different cold temperature conditions or the difference between the maximum and minimum LTE values.

The percentages 10%, 50%, and 90% were interpreted as indicative of minor, moderate, and severe damage, or damage levels, respectively, caused by these temperature variations.

- *CH_base_* was the baseline cold hardiness level of grapevine buds (corresponding to *X_a_* ).
- Δ*CH* represented the change in cold hardiness due to cold stress (corresponding to *X_b_* ).
- *CH_adjusted_* represented the adjusted cold hardiness level after exposure to cold stress.

Accordingly, the adjusted cold hardiness levels post-exposure to cold stress were modeled using the following formulations:

**Minor stress impact**: The adjusted cold hardiness level after a minor damage impact, calculated as 10% of the change induced by the cold stress, was given by:


$$CHminor=\left(\frac{\text{Δ}CH\times 10}{100}\right)+\;CHbase$$

This formula aimed to quantify the hardiness of the buds when subjected to minimal cold stress and provided insight into their initial response to cold temperatures.

**Moderate stress impact**: The adjusted cold hardiness level after a moderate damage impact, calculated as 50% of the change induced by the cold stress, was given by:


$$CHmoderate=\left(\frac{\text{Δ}CH\times 50}{100}\right)+\;CHbase$$

This calculation sheds light on understanding the moderate adaptation of grapevine buds to increasing levels of cold damage.

**Severe stress impact**: The adjusted cold hardiness level after a severe damage impact, calculated as 90% of the change induced by the cold stress, was given by:


$$CHsevere=\left(\frac{\text{Δ}CH\times 90}{100}\right)+\;CHbase$$

This calculation was critical for assessing the upper limits of grapevine bud hardiness to extreme cold conditions.

Comprehensive statistical analyses, including both the two-way ANOVA and Duncan’s multiple range test, were executed utilizing the Statistical Analysis System (SAS) software (version 9.4, SAS Institute, Cary, NC, USA, 2002). Upon identifying significant effects through the two-way ANOVA, Duncan’s multiple range test was employed as a *post-hoc* analysis to further elucidate the specific group differences. The statistical analysis of these LTE values was conducted using a two-way analysis of variance (ANOVA) to discern any significant differences attributable to factors such as bud position along the cane. For the purposes of this study, a p-value threshold of less than 0.05 was established as the criterion for statistical significance.

## Results

In our study, climate data for the months of September and January were followed owing to both the initiation of acclimation in vines when air temperatures fall below 10°C and the critical temperatures of -1.1, -9.4 and -17.8°C, which could lead to frost or cold damage in the buds during autumn. It was observed that the daily temperature change in September was higher than in other months, the maximum temperature reached 34.5°C and the minimum temperature decreased to 3.2°C. The largest temperature difference between night (3.2°C) and day (23.3°C) was 20.1°C. After October 4^th^, temperatures significantly decreased, with nine days when minimum temperatures fell below 0°C. The air temperature reached -0.9°C on October 7^th^, and the first samples for testing at -1.1°C were collected on October 8^th^. In November, minimum temperatures were consistently below 0°C, and samples for testing at -9.4°C were collected the day after November 23^rd^, when the air temperature reached -14.7°C. The coldest night of the month occurred on November 27^th^, with the temperature dropping to -19.4°C. Although the air temperature dropped to the targeted -17.8°C four days later, samples were not collected from the cultivars, because it was possible to determine whether there had been any damage to the buds by collecting samples just before the mid-winter minimum temperatures, when the air temperature had already dropped to 0°F (-17.8°C). Therefore, to obtain clearer information on potential cold damage after the exposure to variable cold conditions during autumn, the final samples were collected just before the onset of winter’s minimum temperatures, on 9 January when the air temperature had dropped to -17.8°C (the air temperature was -18.2°C on January 8^th^). Thus, the rates of damage to grape buds due to fluctuating low temperatures occurring up to January 8^th^, as well as the LTE threshold values, were determined. In December, the air temperatures were quite low, dropping to -16.3°C on December 18^th^ (**Figure 1**).

### Comparative cold hardiness of grape cultivars according to varying temperatures and vine ages

In our study, the cold hardiness of four grape cultivars (‘Itasca’, ‘Frontenac’, ‘La Crescent’, and ‘Marquette’) was assessed at two vine ages (eight-year-old and one-year-old) by determining the lethal temperatures (LT_10_, LT_50_, and LT_90_) that cause 10%, 50%, and 90% bud mortality, respectively, at a sampling temperature of -1.1°C, -9.4°C and -17.8°C. At -1.1°C, ‘Frontenac’ exhibited the greatest cold hardiness among the eight-year-old vines, with LT values of -14.4°C (LT_10_), -18.4°C (LT_50_), and -22.4°C (LT_90_). ‘La Crescent’ was the least cold hardy, with significantly higher LT_50_ and LT_90_ values compared to ‘Frontenac’. ‘Itasca’ and ‘Marquette’ displayed intermediate cold hardiness, with LT values that did not significantly differ from ‘La Crescent’ at LT_10_, while comparable to ‘Frontenac’ at LT_50_ and LT_90_. The p-values (0.254 for LT_10_, 0.096 for LT_50_, and 0.078 for LT_90_) suggested that there were no statistically significant differences at LT_10_, but that there were trends towards significance at LT_50_ and LT_90_. For one-year-old vines, ‘Frontenac’ again showed greater cold hardiness at LT_10_ and LT_50_ compared to ‘Itasca’ and ‘La Crescent’, with ‘Marquette’ exhibiting intermediate values. Statistical analysis revealed significant differences among cultivars at LT_10_ and LT_50_ but not at LT_90_. ‘Itasca’ and ‘La Crescent’ had significantly lower cold hardiness at LT_10_ and LT_50_ compared to ‘Frontenac’. The p-values for one-year-old vines (0.022 for LT_10_, 0.033 for LT_50_, and 0.256 for LT_90_) indicated significant differences among cultivars at LT_10_ and LT_50_, but not at LT_90_. On the other hand, for the eight-year-old vines, at -9.4°C, the LT values indicated similar levels of cold hardiness across all cultivars, with LT_10_ values ranging from -15.8°C to -16.4°C, LT_50_ values from -19.6°C to -21.1°C, and LT_90_ values from -23.4°C to -25.9°C. ‘Frontenac’ displayed a marginally higher cold hardiness at LT_50_ and LT_90_ compared to the other cultivars, though the differences were not statistically significant, as reflected by the p-values (0.946 for LT_10_, 0.549 for LT_50_, and 0.296 for LT_90_). Similarly, the p-values for one-year-old vines (0.391 for LT_10_, 0.419 for LT_50_, and 0.314 for LT_90_) did not show significant statistical differences among the cultivars. The LT values of buds were generally higher (indicating less cold hardiness) than those of the eight-year-old vines, with ‘Frontenac’ demonstrating the lowest cold hardiness (highest LT values) among the younger vines. LT_10_ values ranged from -12.2°C to -14.0°C, LT_50_ from -14.7°C to -17.4°C, and LT_90_ from -17.3°C to -20.9°C. At a sampling temperature of -17.8°C, the p-values (0.814 for LT_10_, 0.747 for LT_50_, and 0.527 for LT_90_) indicated that the differences in cold hardiness among the cultivars were not statistically significant. The LT values for the eight-year-old vines suggested that all cultivars exhibited a degree of cold hardiness, with LT_10_ values ranging from -18.6°C to -19.6°C, LT_50_ from -22.3°C to -23.9°C, and LT_90_ from -25.3°C to -28.3°C. ‘La Crescent’ showed slightly higher LT_50_ and LT_90_ values. Similarly, the p-values (0.450 for LT_10_, 0.368 for LT_50_, and 0.319 for LT_90_) again showed no significant statistical differences among the cultivars. For the one-year-old vines, the LT values were generally closer together. The LT_10_ values ranged from -17.9°C to -19.0°C, LT_50_ from -18.9°C to -20.0°C, and LT_90_ from -19.8°C to -21.0°C. Notably, the one-year-old ‘Frontenac’ vines exhibited the lowest LT values (**Table 1**).

**Table 1:** Comparative cold hardiness of grapevine cultivars at varying temperatures (°C) and vine ages; LT_10_, LT_50_, and LT_90_ indicators.

Sampling time	Vine age	Cultivar	LT_10_	LT_50_	LT_90_
30°F (-1.1°C)	Eight-year-old	Itasca	-13.9 ± 1.0^ns^	-17.2 ± 0.8ab	-20.4 ± 1.1ab
		Frontenac	-14.4 ± 0.8	-18.4 ± 0.8a	-22.4 ± 0.7a
		La Crescent	-12.2 ± 0.9	-15.4 ± 0.9b	-18.7 ± 1.1b
		Marquette	-14.7 ± 0.8	-17.4 ± 0.6ab	-20.1 ± 0.4ab
		*p-value*	0.254	0.096	0.078
	One-year-old	Itasca	-12.3 ± 0.7b	-14.6 ± 0.5b	-16.9 ± 0.3^ns^
		Frontenac	-14.5 ± 0.5a	-16.7 ± 0.7a	-19.0 ± 1.3
		La Crescent	-11.3 ± 0.8b	-13.9 ± 0.6b	-16.5 ± 0.8
		Marquette	-12.9 ± 0.2ab	-15.5 ± 0.6ab	-18.1 ± 0.9
		*p-value*	0.022	0.033	0.256
15°F (-9.4°C)	Eight-year-old	Itasca	-16.4 ± 0.7^ns^	-20.0 ± 0.9^ns^	-23.6 ± 1.1^ns^
		Frontenac	-16.3 ± 0.1	-21.1 ± 0.6	-25.9 ± 1.2
		La Crescent	-15.8 ± 1.1	-19.6 ± 1.0	-23.4 ± 1.0
		Marquette	-16.4 ± 0.9	-20.9 ± 0.8	-25.3 ± 0.8
		*p-value*	0.946	0.549	0.296
	One-year-old	Itasca	-12.9 ± 0.2^ns^	-16.5 ± 0.3^ns^	-20.1 ± 0.5^ns^
		Frontenac	-12.2 ± 0.6	-14.7 ± 0.8	-17.3 ± 1.1
		La Crescent	-13.8 ± 1.2	-17.4 ± 1.4	-20.9 ± 1.7
		Marquette	-14.0 ± 0.9	-16.2 ± 1.4	-18.4 ± 1.9
		*p-value*	0.391	0.419	0.314
0°F (-17.8°C)	Eight-year-old	Itasca	-18.6 ± 0.8^ns^	-23.0 ± 0.8^ns^	-27.5 ± 1.1^ns^
		Frontenac	-19.3 ± 1.0	-22.3 ± 0.8	-25.3 ± 0.7
		La Crescent	-19.6 ± 0.9	-23.9 ± 0.9	-28.3 ± 1.1
		Marquette	-19.5 ± 0.8	-22.8 ± 0.6	-26.0 ± 0.4
		*p-value*	0.814	0.747	0.527
	One-year-old	Itasca	-19.0 ± 0.7^ns^	-20.0 ± 0.5^ns^	-21.0 ± 0.3^ns^
		Frontenac	-17.9 ± 0.5	-18.9 ± 0.7	-19.8 ± 1.3
		La Crescent	-18.1 ± 0.8	-19.1 ± 0.6	-20.0 ± 0.8
		Marquette	-18.9 ± 0.2	-19.6 ± 0.6	-20.2 ± 0.9
		*p-value*	0.450	0.368	0.319

Data is expressed as a means of the data ± SE. For a given factor and significance (p< 0.05), different letters within a column represent significant differences (Duncan test, p< 0.05). ns; not significant.

### Comparative assessment of lethal temperatures across grape cultivars based on vine age (eight-year-old and one-year-old) at different sampling temperatures

**Figure 4:**
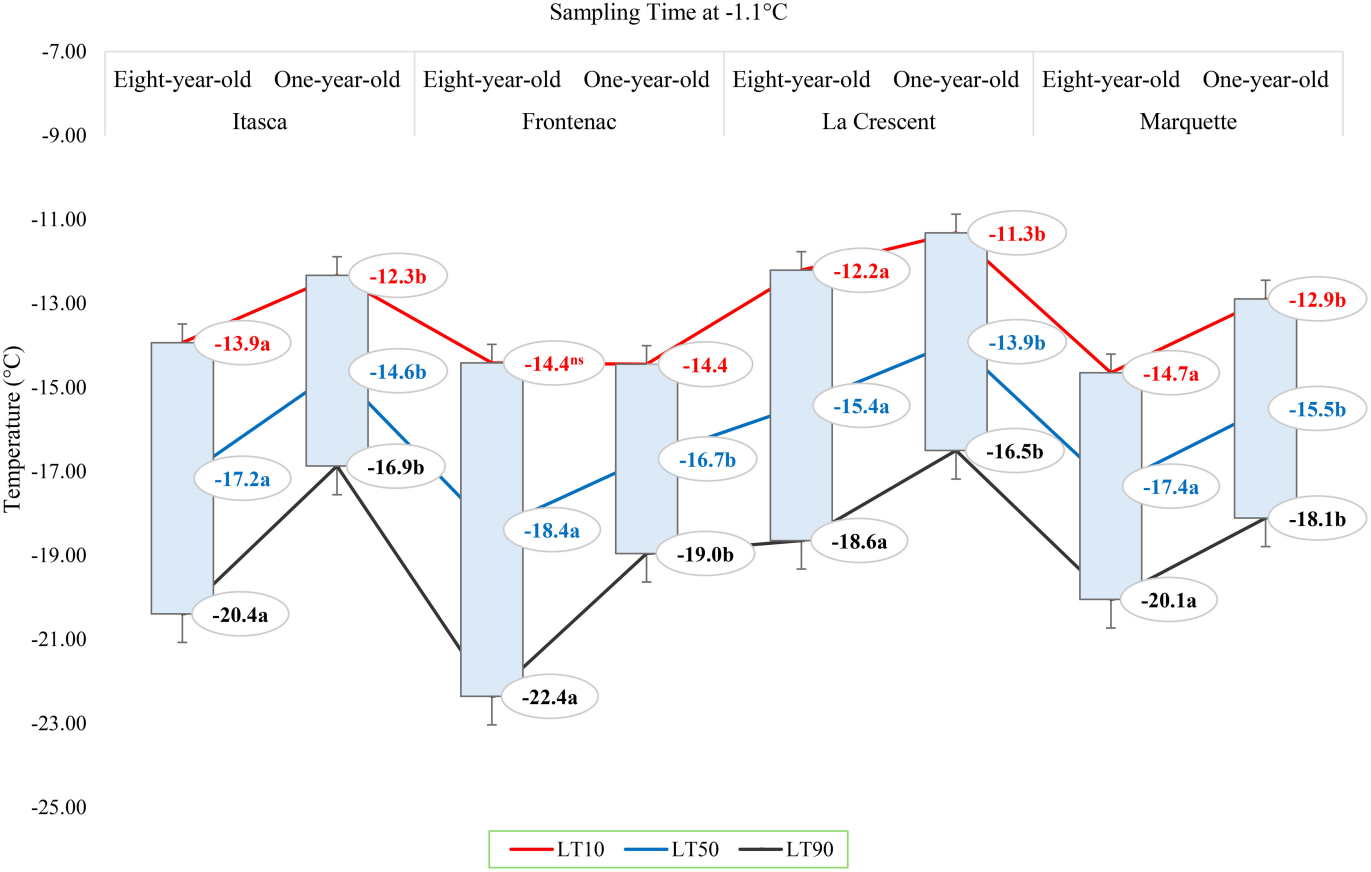
The lethal temperatures (LT_10_, LT_50_, and LT_90_) for grapevine cultivars ‘Itasca’, ‘Frontenac’, ‘La Crescent’, and ‘Marquette’ at two vine ages (eight-year-old and one-year-old), sampled at -1.1°C.

**Figure 5:**
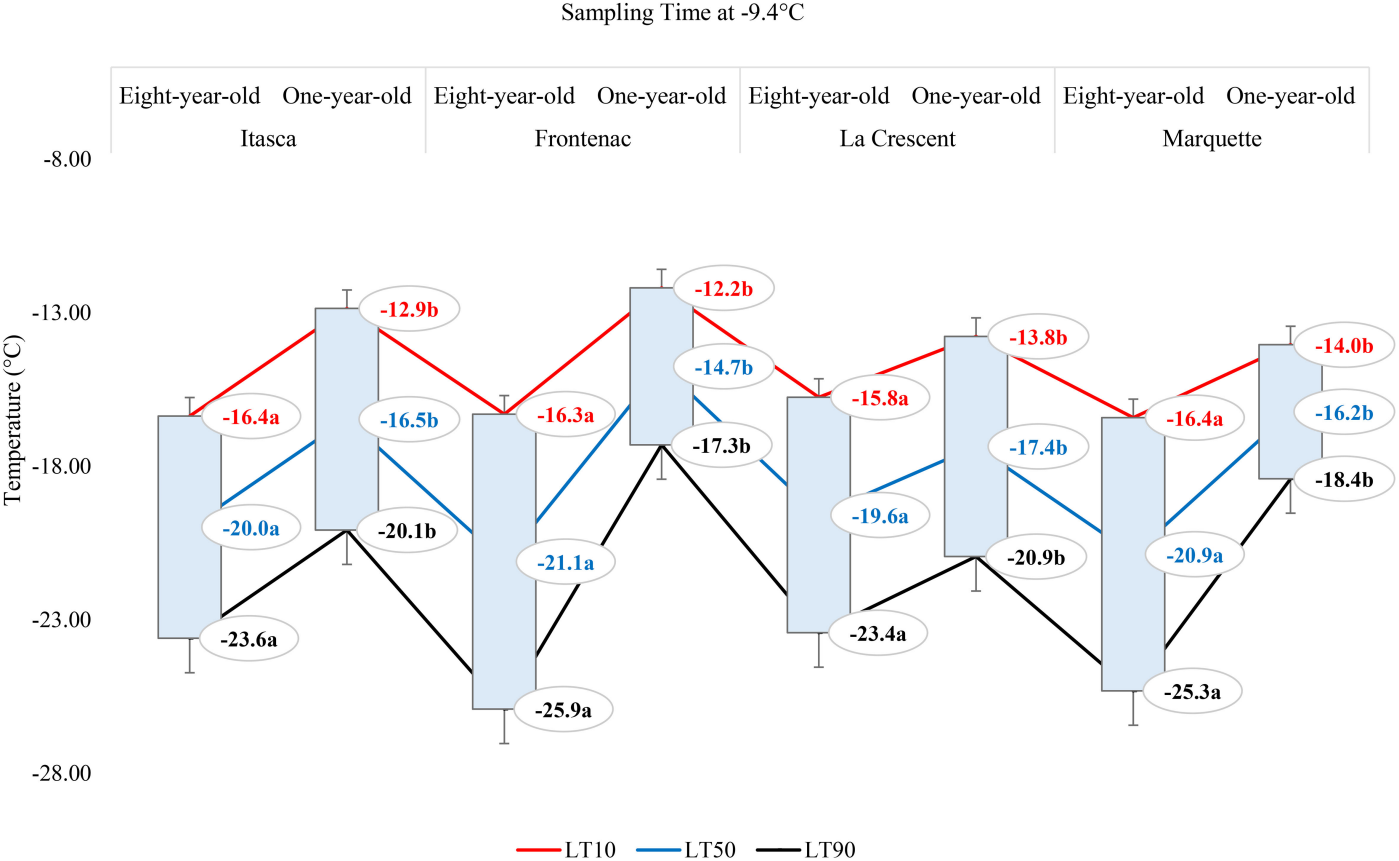
The lethal temperatures (LT_10_, LT_50_, and LT_90_) for grapevine cultivars ‘Itasca’, ‘Frontenac’, ‘La Crescent’, and ‘Marquette’ at two vine ages (eight-year-old and one-year-old), sampled at -9.4°C.

**Figure 6:**
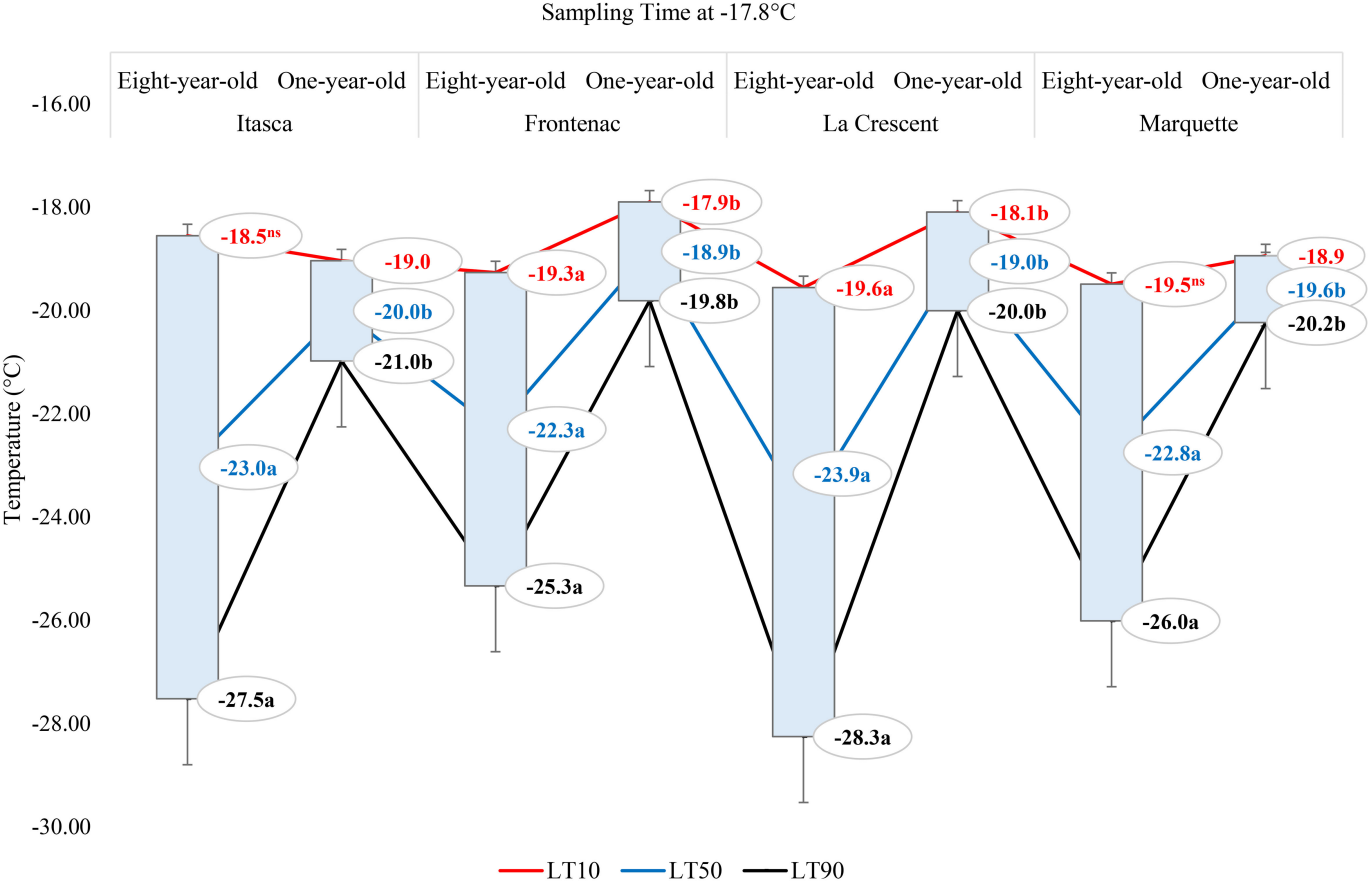
The lethal temperatures (LT_10_, LT_50_, and LT_90_) for grapevine cultivars ‘Itasca’, ‘Frontenac’, ‘La Crescent’, and ‘Marquette’ at two vine ages (eight-year-old and one-year-old), sampled at -17.8°C.

The lethal temperatures (LT_10_, LT_50_, and LT_90_) at which 10%, 50%, and 90% of the buds were dead, respectively, at a sampling temperature of -1.1°C indicated significant differences among the cultivars in terms of LT values, except for the LT_10_ values for ‘Frontenac’ (**Figure 4**). When comparing eight-year-old grapevines to one-year-old vines, the LT values were identified at lower temperatures for the older vines. For the cultivar ‘Itasca’, eight-year-old vines exhibited LT values at significantly lower temperatures compared to one-year-old vines, with differences of 1.6°C for LT_10_, 2.6°C for LT_50_, and 3.5°C for LT_90_. Similarly, ‘Frontenac’ showed a marked increase in cold tolerance with age, evidenced by a difference of 0.1°C at LT_10_, 1.7°C at LT_50_, and 3.4°C at LT_90_ between eight-year-old and one-year-old vines. The cultivar ‘La Crescent’ revealed a differential cold hardiness, with the eight-year-old vines demonstrating a 0.9°C lower LT_10_, 1.5°C lower LT_50_, and 2.1°C lower LT_90_ than the one-year-old counterparts. ‘Marquette’ showed LT values that were 1.8°C lower at LT_10_, 1.9°C lower at LT_50_, and 2°C lower at LT_90_ for the eight-year-old vines in comparison to those one-year-old. Upon analyzing LTE values at -9.4°C, a discernible pattern emerged showing differences in cold hardiness between vines of different ages. Eight-year-old ‘Itasca’ vines were observed to exhibit slightly higher cold hardiness than their one-year-old counterparts, with LT_10_, LT_50_ and LT_90_ threshold values being 3.5°C lower, respectively. For ‘Frontenac’ there was a significant increase in cold tolerance with age of the vine, whereas eight-year-old vines showed lower temperatures of 4.1°C for LT_10_, 6.4°C for LT_50_ and 8.6°C for LT_90_. The ‘La Crescent’ cultivar showed difference in LT values between vine ages, and the older vines were colder hardy at LT_10_, LT_50_ and LT_90_ thresholds by 2.0°C 2.2°C and 2.5°C, respectively. ‘Marquette’ vines displayed a similar trend, with eight-year-old vines showing more cold-hardiness by 2.4°C for LT_10_, 4.7°C for LT_50_, and 6.9°C for LT_90_ compared to one-year-old vines. These results collectively indicated that older vines, across these cultivars, tend to have increased cold hardiness, especially at the more critical LT_50_ and LT_90_ thresholds (**Figure 5**). Considering data for grapevine cultivars sampled at 0°F (-17.8°C), there was generally a trend of increased cold hardiness in older vines, particularly noticeable in the LT_50_ and LT_90_ values, where the eight-year-old vines often showing greater hardiness to cold compared to the one-year-old vines. Our results indicated that eight-year-old ‘Itasca’ vines exhibited LT values that were consistently lower than those of their one-year-old counterparts, a substantial 3.0°C difference at LT_50_, and an even more pronounced 6.5°C difference at LT_90_, except for LT_10_ values. ‘Frontenac’ followed a similar trend, where the eight-year-old vines’ LT_10_, LT_50_ and LT_90_ values were 1.4°C, 3.4 and 5.5°C, respectively, lower than that of the one-year-old vines. For ‘La Crescent’, a pronounced increase in cold hardiness with vine age was evident, with the eight-year-old vines demonstrating lower temperatures by 1.5°C for LT_10_, 4.9°C for LT_50_, and 8.3°C for LT_90_. ‘Marquette’ vines presented a nuanced profile, with a minor 0.6°C difference at LT_10_, a significant 3.2°C difference at LT_50_, and a 5.8°C difference at LT_90_ (**Figure 6**).

### Comparison of the change in LT_50_ temperatures for buds in different grape cultivars at three sampling temperatures

Our research documented LT_50_ values for the grapevine cultivars ‘Itasca’, ‘Frontenac’, ‘La Crescent’, and ‘Marquette’ at three sampling temperatures: -1.1°C, -9.4°C, and -17.8°C. Our results showed that there were significant differences between cultivars in three separate sampling periods for all buds. For ‘Itasca’ cultivar at -1.1°C, the LT_50_ values showed a range from -15.6°C in bud-3 to -21.8°C in bud-8. The highest cold vulnerability within this cultivar were determined in buds-8 and buds-9, with LT_50_ values of -21.8°C and -20.2°C, respectively. The ‘Frontenac’ showed less variation in LT_50_ values, spanning from -18.2°C in buds-2 and buds-3 to -20.4°C in buds-7, with buds-1 and buds-7 presenting the highest cold hardiness. The buds-2 and buds-3 had a significantly different LT_50_ value from the others. The LT_50_ values of ‘La Crescent’ varied more widely, from -14.2°C in buds 1 to -20.0°C in buds-8. The buds-7 and buds-8 of this cultivar’s were the hardiest to cold conditions, with LT_50_ values of -18.2°C and -20.0°C, respectively. The statistical analysis indicated a significant difference in cold hardiness, particularly in buds-8. The LT_50_ values of the ‘Marquette’ cultivar ranged from -15.6°C in buds-5 to -20.5°C in buds-6. The buds-3 and buds-6 of this cultivar’s were observed to be the least cold-hardy, with LT_50_ values of -19.3°C and -20.5°C, respectively. Considering sampling time at -9.4°C, the variance in cold hardiness occurred more pronounced. ‘Frontenac’ showed considerable hardiness, especially at buds-9 with an LT_50_ value of -30.2°C, the greatest cold-hardiness observed at this temperature across all cultivars. ‘La Crescent’ also displayed high cold-hardiness in buds-9 at -30.0°C. ‘Itasca’ and ‘Marquette’ were generally more sensitive, with lower LT_50_ values. The temperature difference was stark between ‘Frontenac’ and ‘La Crescent’ for buds-9, at 0.2°C. Regarding -17.8°C, ‘La Crescent’ showed significant cold hardiness, particularly in buds-8 and buds-9, with LT_50_ values of -29.9 and -27.4°C, respectively. ‘Frontenac’ and ‘Marquette’ exhibited mixed responses, while ‘Itasca’ was generally more sensitive, with lower LT_50_ values across its buds. The temperature difference between the hardiest (‘La Crescent’) and the most sensitive (‘Itasca’) cultivars was notable, especially in buds-8, where it reached 8.9°C (**Table 2**). On the other hand, our research evaluated the bud death rates for four grape cultivars -’Itasca’, ‘Frontenac’, ‘La Crescent’, and ‘Marquette’ - across three sampling temperatures: -1.1°C, -9.4°C, and -17.8°C. At -1.1°C, the bud death rates were predominantly zero across all cultivars. However, a notable exception was observed in ‘Itasca’, where buds-7 experienced a 5% death rate. Moving to -9.4°C, the pattern of minimal bud death continued, with most buds across all cultivars showing no death. Exceptions were found in ‘Itasca’ (buds-3), Frontenac (buds-4), ‘La Crescent’ (buds-7), and ‘Marquette’ (buds-5), each recording a 5% death rate. At the coldest temperature of -17.8°C, again, most buds across all cultivars exhibited no death. The exceptions were ‘Itasca’ (buds-8), ‘Frontenac’ (buds-4), ‘La Crescent’ (buds-6), and ‘Marquette’ (buds-3), each with a 5% death rate. This indicated a consistent pattern of hardiness across the cultivars, with only isolated instances of vulnerability (**Table 3**).

**Table 2:** Variability in LT_50_ temperatures (°C) for buds across different grapevine cultivars at three sampling temperatures.

LT_50_ Values										
Sampling time	Cultivar	Buds-1	Buds-2	Buds-3	Buds-4	Buds-5	Buds-6	Buds-7	Buds-8	Buds-9
30°F (-1.1°C)	Itasca	-16.2 ± 1.5b	-17.8 ± 0.6a	-15.6 ± 0.5b	-16.7 ± 0.8ab	-18.8 ± 1.9a	-16.6 ± 1.0b	-17.2 ± 0.8b	-21.8 ± 1.3a	-20.2 ± 1.1a
	Frontenac	-19.6 ± 1.3a	-18.2 ± 1.0a	-18.2 ± 1.3ab	-18.6 ± 0.9a	-18.9 ± 0.7a	-19.0 ± 1.0ab	-20.4 ± 0.9a	-19.4 ± 1.2ab	-19.4 ± 1.1ab
	La Crescent	-14.2 ± 0.8b	-16.1 ± 0.7b	-16.5 ± 1.0b	-15.4 ± 1.2b	-15.2 ± 1.0b	-17.1 ± 0.7b	-18.2 ± 1.3ab	-20.0 ± 1.2ab	-16.5 ± 0.7b
	Marquette	-17.2 ± 0.6ab	-16.4 ± 0.8b	-19.3 ± 0.7a	-17.8 ± 0.9ab	-15.6 ± 0.8b	-20.5 ± 1.0a	-17.9 ± 0.6ab	-17.7 ± 1.2b	-19.2 ± 1.0ab
	*p value*	0.106	0.025	0.105	0.0.40	0.079	0.171	0.006	0	0
15°F (-9.4°C)	Itasca	-18.7 ± 1.4c	-22.2 ± 1.1a	-17.6 ± 0.7b	-20.9 ± 1.0a	-19.5 ± 1.6ab	-19.1 ± 1.1b	-19.1 ± 0.8b	-21.0 ± 0.9b	-18.8 ± 1.6b
	Frontenac	-23.5 ± 2.0a	-22.5 ± 1.1a	-21.0 ± 1.3a	-20.3 ± 1.0a	-21.7 ± 0.8a	-22.8 ± 0.9a	-16.4 ± 0.6b	-27.7 ± 0.7a	-30.2 ± 0.6a
	La Crescent	-21.8 ± 1.1b	-17.9 ± 1.0b	-18.7 ± 1.0ab	-16.8 ± 1.4b	-17.8 ± 1.5b	-17.8 ± 1.2b	-24.4 ± 1.8a	-25.1 ± 1.8a	-30.0 ± 0.4a
	Marquette	-18.6 ± 1.5c	-21.1 ± 1.7ab	-19.3 ± 1.1ab	-21.2 ± 1.0a	-22.3 ± 0.8a	-20.5 ± 1.1ab	-23.7 ± 0.8a	-21.3 ± 0.4b	-21.1 ± 1.2b
	*p value*	0.09	0.062	0.034	0.056	0.023	0	0	0	0
0°F (-17.8°C)	Itasca	-21.4 ± 1.0b	-24.0 ± 1.0a	-19.7 ± 1.1b	-23.0 ± 0.9a	-21.9 ± 1.1ab	-20.9 ± 0.7b	-24.5 ± 0.9a	-21.0 ± 0.8c	-23.0 ± 1.0b
	Frontenac	-25.2 ± 0.8a	-22.4 ± 1.2ab	-20.3 ± 1.0b	-19.3 ± 0.7b	-20.6 ± 0.5b	-19.8 ± 1.0b	-25.5 ± 0.9a	-23.0 ± 0.6b	-23.5 ± 0.9b
	La Crescent	-23.1 ± 1.3ab	-23.9 ± 1.1a	-23.2 ± 1.4a	-23.1 ± 1.4a	-24.6 ± 1.2a	-22.7 ± 0.9a	-26.3 ± 0.8a	-29.9 ± 0.5a	-27.4 ± 1.5a
	Marquette	-23.0 ± 1.0ab	-20.0 ± 0.5b	-23.1 ± 1.4a	-22.7 ± 1.0a	-21.8 ± 1.3ab	-22.1 ± 1.1a	-22.0 ± 0.8b	-20.7 ± 1.1c	-19.1 ± 0.8c
	*p value*	0.014	0.176	0.029	0.135	0.056	0.027	0.121	0.151	0.056

Data is expressed as a means of the data ± SE. For a given factor and significance (p< 0.05), different letters within a column represent significant differences (Duncan test, p< 0.05).

**Table 3:** Percentage rates of bud death in different grapevine cultivars at three sampling temperatures.

Bud death rate (%)										
Sampling time	Cultivar	Buds-1	Buds-2	Buds-3	Buds-4	Buds-5	Buds-6	Buds-7	Buds-8	Buds-9
30F (-1.1°C)	Itasca	0.0	0.0	0.0	0.0	0.0	0.0	5.0	0.0	0.0
	Frontenac	0.0	0.0	0.0	0.0	5.0	0.0	0.0	0.0	0.0
	La Crescent	0.0	0.0	5.0	0.0	0.0	0.0	0.0	0.0	0.0
	Marquette	0.0	0.0	0.0	0.0	0.0	5.0	0.0	0.0	0.0
15F (-9.4°C)	Itasca	0.0	0.0	5.0	0.0	0.0	0.0	0.0	0.0	0.0
	Frontenac	0.0	0.0	0.0	5.0	0.0	0.0	0.0	0.0	0.0
	La Crescent	0.0	0.0	0.0	0.0	0.0	0.0	5.0	0.0	0.0
	Marquette	0.0	0.0	0.0	0.0	5.0	0.0	0.0	0.0	0.0
0F (-17.8°C)	Itasca	0.0	0.0	0.0	0.0	0.0	0.0	0.0	5.0	0.0
	Frontenac	0.0	0.0	0.0	5.0	0.0	0.0	0.0	0.0	0.0
	La Crescent	0.0	0.0	0.0	0.0	0.0	5.0	0.0	0.0	0.0
	Marquette	0.0	0.0	5.0	0.0	0.0	0.0	0.0	0.0	0.0

### Comparison of LT_50_ temperature values according to the positions of the buds on the nodes of each grape cultivar

**Figure 7:**
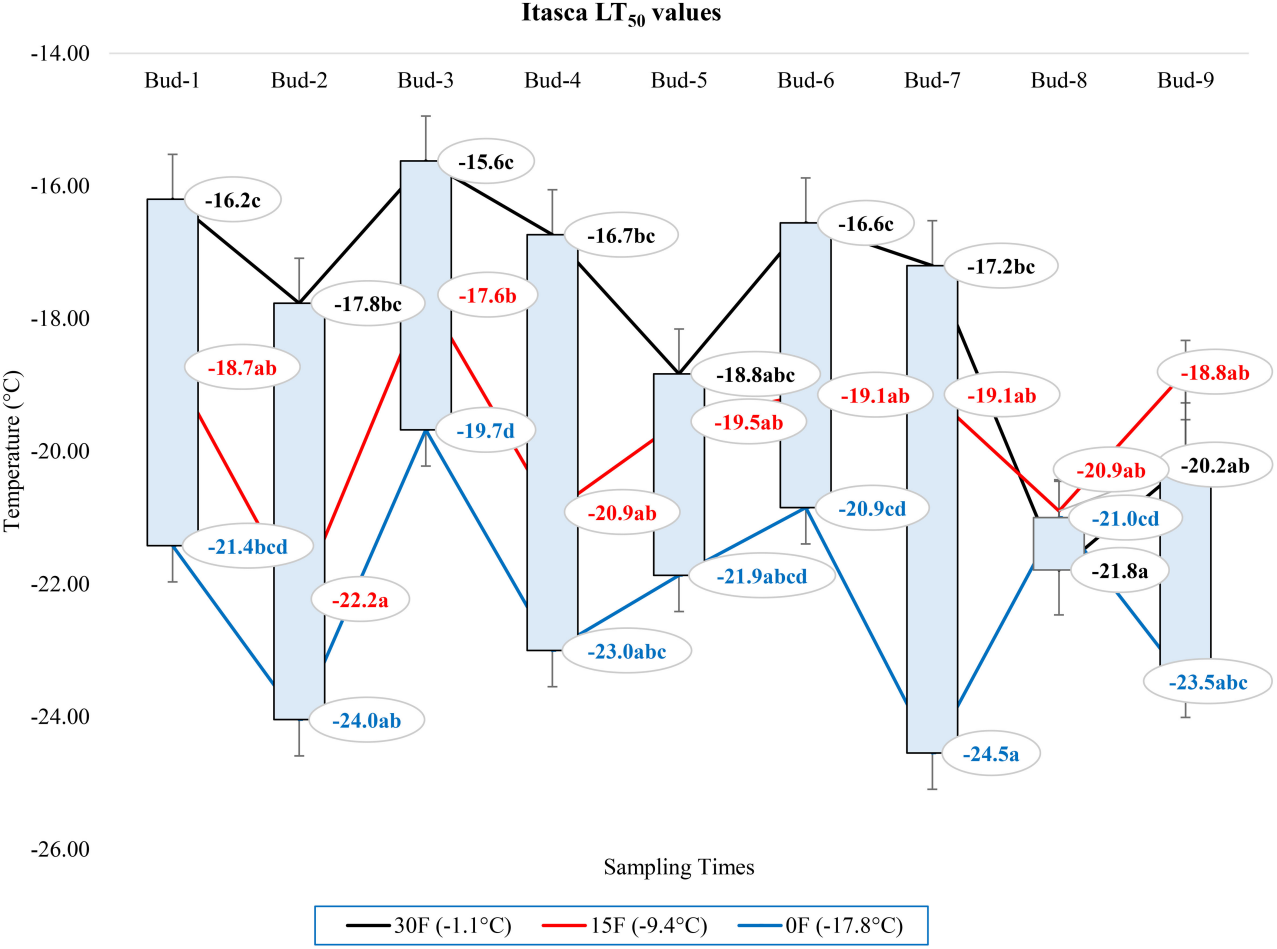
Distribution of LT_50_ temperature values for (°C) ‘Itasca’ grapevine buds at varying sampling times.

**Figure 8:**
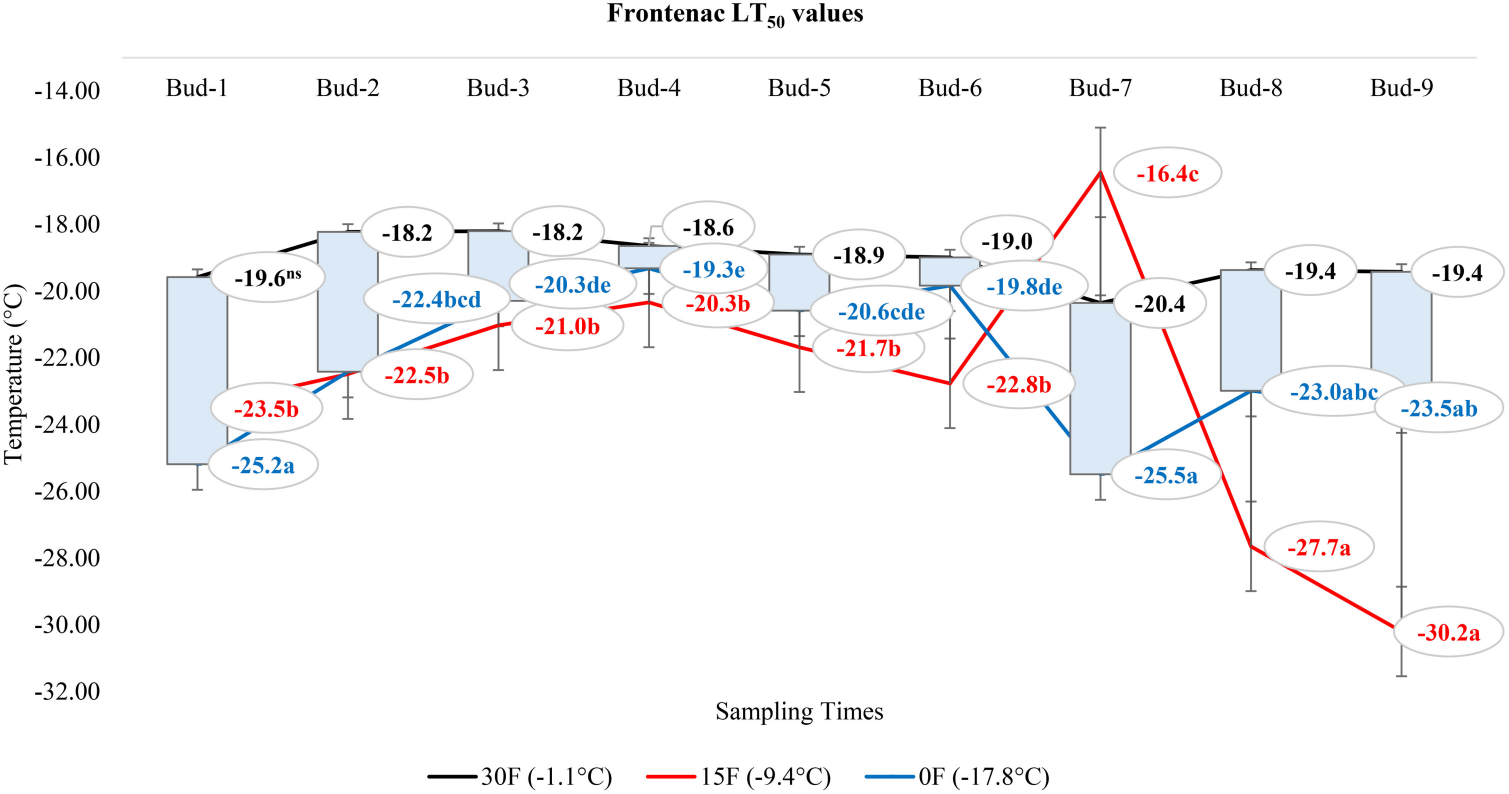
Distribution of LT_50_ temperature values (°C) for ‘Frontenac’ grapevine buds at varying sampling times.

**Figure 9:**
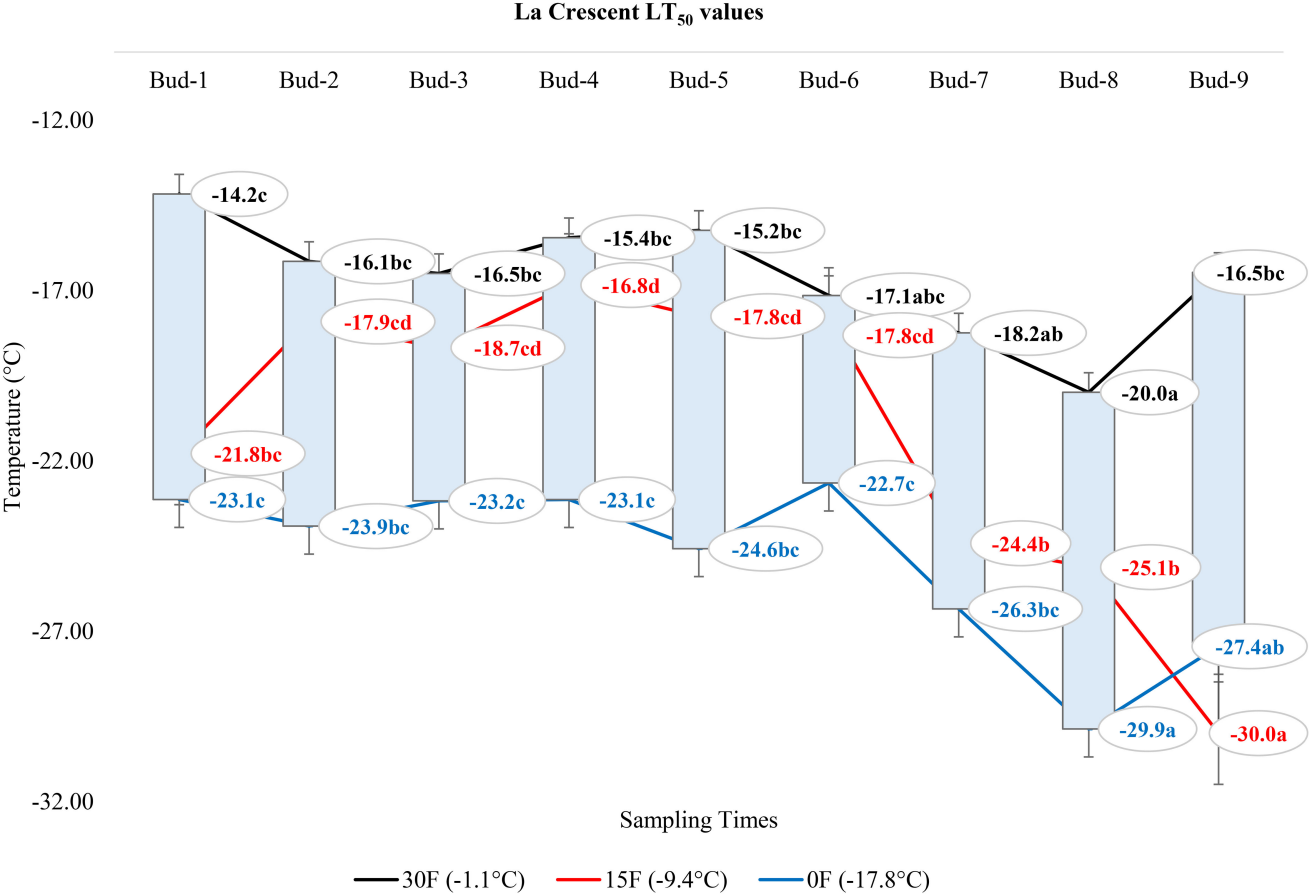
Distribution of LT_50_ temperature values (°C) for ‘La Crescent’ grapevine buds at varying sampling times.

**Figure 10:**
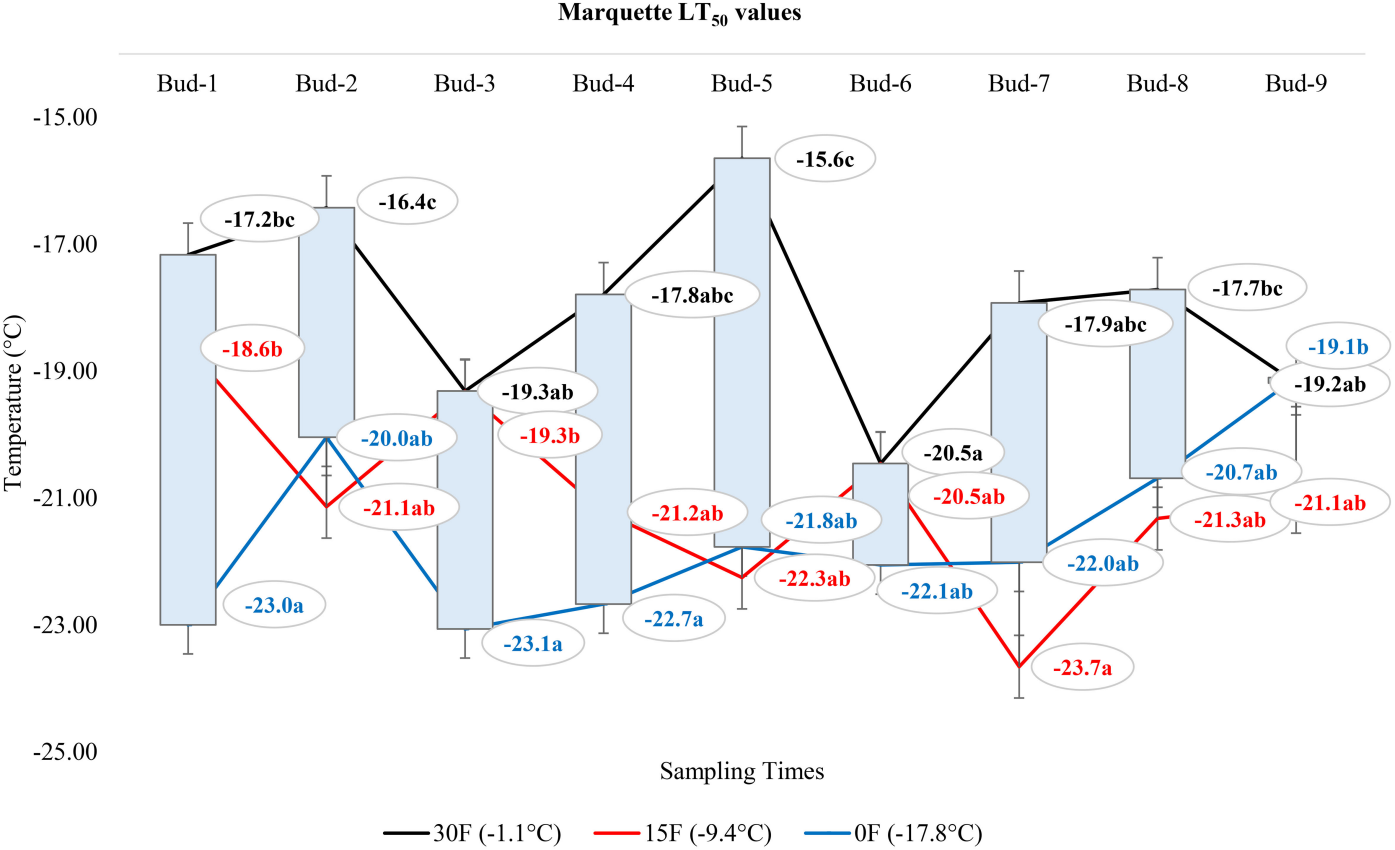
Distribution of LT_50_ temperature values (°C) for ‘Marquette’ grapevine buds at varying sampling times.

At -1.1°C) for ‘Itasca’ cultivar, the coldest-hardy bud was buds-8, followed by buds-5 and buds-2, which showed LT_50_ values of -18.8°C and -17.8°C, respectively. On the other end of the spectrum, the most sensitive bud was buds-3, with an LT_50_ value of -15.6°C, closely followed by buds-1 and buds-6, which had LT_50_ values of -16.2°C and -16.6°C. At 15°F (-9.4°C), buds-2 demonstrated the highest hardiness with an LT_50_ value of -22.2°C, and subsequent hardiness were buds-8 and buds-5, with LT_50_ values of -20.9°C and -20.9°C. The most sensitive bud at this temperature was buds-3, with an LT_50_ of -17.6°C, with buds-6 and buds-7 also showing sensitivity with LT_50_ values of -19.1°C each. When the temperature dropped to -17.8°C, buds-7 emerged as the most hardiness with an LT_50_ of -24.5°C. Following in hardiness were buds-2 and buds-9 with LT_50_ values of -24.0°C and -23.5°C, respectively. The most sensitive were buds-3 at -19.7°C, with buds-7 and buds-8 exhibiting more sensitivity with LT_50_ values of -21.9°C and -21.0°C, respectively (**Figure 7**). Regarding the LT_50_ values for the ‘Frontenac’ cultivar at -1.1°C, there was no difference between LT values; however, the coldest-hardy bud was buds-7 with an LT_50_ value of -20.4°C. Following in hardiness were buds-1, buds-8, and buds-9, with LT_50_ values of -19.6, -19.4°C, respectively. The most sensitive buds were buds-2 and buds-3, with an LT_50_ of -18.2°C, and buds-4 followed it in sensitivity, with an LT_50_ value of -18.6°C. At -9.4°C, buds-9 showed the highest hardiness with an LT_50_ of -22.5°C, and buds-8 was next, with an LT_50_ of -27.7°C. There was no statistical difference between other buds. When temperatures dropped to -17.8°C, the most hardiness bud was buds-9, with an LT_50_ of -30.2°C, indicating significant cold hardiness. Buds-7 was the next hardest with an LT_50_ of -25.5°C. The most sensitive at this temperature were buds-3, buds-4, buds-5 and buds-6, with an LT_50_ of -20.3, -19.3, -20.6 and -19.8°C, respectively (**Figure 8**). Considering the LT_50_ values for the ‘La Crescent ‘ cultivar at -1.1°C, buds-1 was the most sensitive with an LT_50_ value of -14.2°C, closely followed by buds-2, buds-3, buds-4, buds-5 buds-6, and buds-9. The coldest-hardy at this temperature was buds-8 with an LT_50_ of -20.0°C, with buds-7 and buds-6 next in line, both showing an LT_50_ of -18.2°C and -17.1°C respectively. For the -9.4°C temperature, buds-9 remained the most hardiness with an LT_50_ value of -30.0°C. Subsequently, buds-8 displayed notable hardiness with an LT_50_ of -25.1°C, and buds-7 with -24.4°C. The most sensitive bud at this colder temperature was buds-4, showing an LT_50_ of -16.8°C, but buds-2, buds-3, buds-4, buds-5 and buds-6 had similar LT_50_ values. At the coldest temperature of -17.8°C, buds-8 was identified as the most cold-hardy with an impressive LT_50_ of -29.9°C, while buds-9 was almost equally hardy at -27.4°C. Buds-6 were found to be the most sensitive with an LT_50_ of -22.7°C, and buds-1, buds-2, buds-3, buds-4, buds-5, buds-6 and buds-7 had similar LT_50_ values (**Figure 9**). Our research on the ‘Marquette’ grape cultivar at -1.1°C found that buds-6 was the most cold-hardy with an LT_50_ value of -20.5°C. Buds-3, buds-4, buds-7, and buds-9 followed in cold-hardiness, exhibiting LT_50_ values of -19.3, -17.8°C, -17.9 and -19.2, respectively. Buds-5 emerged as the most sensitive at this temperature, possessing an LT_50_ of -15.6°C, but there was no statistical difference between buds-1, buds-2, buds-4, buds-5, buds-6, buds-7, and buds-8 LT_50_ values. From the -9.4°C sampling, our findings indicated that buds-7 was the coldest-hardy with an LT_50_ of -23.7°C. There was no statistical difference between other buds LT_50_ values, except for buds-1 and buds-3. buds-1 was the most sensitive at this temperature, with an LT_50_ of -18.6°C, followed by bud-3, which had LT_50_ values of -19.3°C. At the lowest sampled temperature of -17.8°C, our results showed that buds-1 and buds-3 were the most cold-hardy with an LT_50_ of -23.0 and -23.1°C, respectively. However, there were no significant differences between the LT_50_ values of the buds except buds-9. In contrast, buds-9 was the most sensitive, with an LT_50_ of -19.1°C (**Figure 10**).

### General evaluation

**Figure 11:**
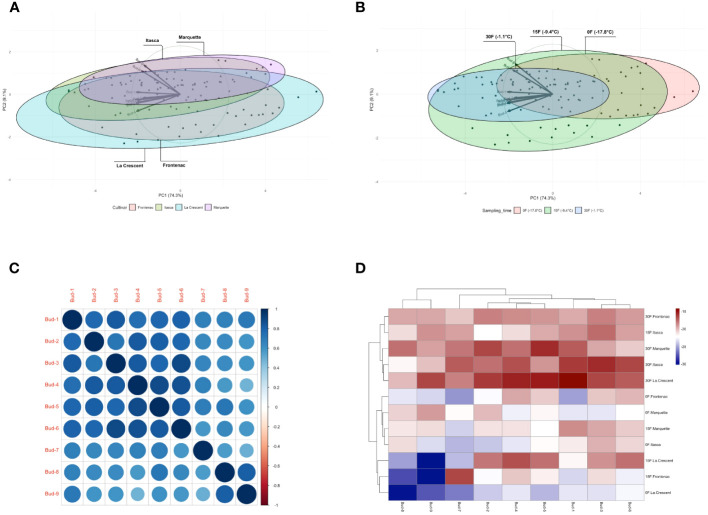
Multivariate and correlation analyses of grapevine cultivar responses at varying temperatures: PCA distribution bud mortality **(A, B)**, Correlation Matrix **(C)** and Heatmaps **(D)**.

Our findings included that the first two PCA biplots showed a distribution of data points for different grape cultivars and sampling temperatures. In the cultivar centric PCA, the ‘Itasca’ and ‘Marquette’ cultivars overlapped significantly. It indicated similarities in their variance, while ‘Frontenac’ and ‘La Crescent’ were more distinct (**Figure 11A**). The PCA biplot for sampling times showed that data points for -17.8°C formed a distinct cluster, while those for -1.1°C and -9.4°C exhibited some overlap (**Figure 11B**). Our results from the correlation matrix indicated that there were strong positive correlations between most of the buds. Specifically, bud-1 was positively correlated with buds-2 and buds-9; buds-2 showed a similar pattern of positive correlation with buds-3 and buds-9, and this pattern was consistent for the subsequent buds in the series. Each bud tended to show a strong positive correlation with the buds listed after it. There were no strong negative correlations observed between any of the buds in the matrix (**Figure 11C**). The heatmap revealed patterns of LT_50_ values across different cultivars and temperatures. Certain groups, like those for Itasca at -17.8°C and Marquette at -9.4°C, showed higher LT_50_ values, which were indicated by the lighter shades. Conversely, darker shades, such as those for ‘Frontenac’ at -1.1°C, represented lower LT_50_ values. This suggested a variability in cold hardiness within and between the cultivars at different temperatures (**Figure 11D**).

## Discussion

Our results indicate a significant diurnal temperature variation in September, with a maximum temperature of 34.5°C and a minimum of 3.2°C, indicating the initial phase of acclimation in grapevines as they respond to decreasing temperatures. This finding is particularly relevant considering the impact of such temperature fluctuations on the physiological processes involved in cold hardiness. The 20.1°C temperature difference observed between day and night during this period indicates that vines must adapt rapidly to prevent damage from frost or cold damage. These results are consistent with the findings of (Howell and Shaulis, 1980), which highlights the critical role of daily temperature changes in grapevines. Additionally, our findings in October showed a significant drop in temperatures, with minimum temperatures falling below 0°C for nine days. This transition is crucial to understanding the acclimation process as it signals that the vines are entering a more sensitive phase. The occurrence of -0.9°C on 7 October and subsequent sampling highlights the importance of monitoring critical temperature thresholds for cold damage in grapevines, which is in line with practices recommended by Mosedale et al. (2015). The consistent sub-zero minimum temperatures in November and the collection of canes following a -14.7°C reading on November 23^rd^ further illustrate the vines’ progression towards deeper dormancy and increased cold hardiness. This stage is critical to assess whether the vines are ready for the harsher conditions of winter, which is consistent with findings that late autumn and early winter temperatures are important in determining the cold hardiness levels of vines (Ferguson et al., 2014). The strategic decision not to collect samples after the air temperature reached -17.8°C (0°F) and our December observations, with temperatures dropping to -16.3°C (**Figure 1**), are informed by the understanding that the most accurate assessment of cold damage potential and acclimation success can be made by evaluating the conditions just before the onset of the minimum winter temperatures. This approach is consistent with results suggesting that assessing bud cold tolerance before winter minimum provides a reliable indicator of vine cold tolerance and/or hardiness (Dami et al., 2005).

### Comparative cold hardiness of grape cultivars according to varying temperatures and vine ages

This research aimed to evaluate the hardiness of these distinct grape cultivars to winter temperatures. In study, we conducted a detailed analysis of the cold hardiness in ‘Itasca’, ‘Frontenac’, ‘La Crescent’, and ‘Marquette’ grape cultivars. The focus was on vines aged one and eight years, examining their survival at LT_10_, LT_50_, and LT_90_ values which correspond to the onset of 10%, 50%, and 90% bud mortality, respectively. These temperatures were detected at three specific cold thresholds: -1.1°C, -9.4°C, and -17.8°C. Our findings show that ‘Frontenac’ displayed superior cold hardiness, especially in eight-year-old vines, as indicated by its lower lethal temperatures (LT_50_ and LT_90_). In contrast, ‘La Crescent’ showed higher susceptibility to cold, with higher LT_50_ and LT_90_ values, aligning with the observations of Ferguson et al. (2014). The ‘Itasca’ and ‘Marquette’ cultivars showed intermediate cold hardiness, with their LT_10_ values being comparable to ‘La Crescent’, but at LT_50_ and LT_90_, they mirrored ‘Frontenac’s’ fortitude. The ‘Frontenac’ cultivar consistently demonstrated superior cold hardiness when we examined one-year-old vines. This was evident in its low LT_10_ and LT_50_ values, which significantly exceeded those of ‘Itasca’ and ‘La Crescent’, highlighting its robustness in young vines. In fact, our observations that ‘Frontenac’ exhibits superior cold tolerance are consistent with the findings of Covert (2011), who indicated the exceptional cold hardiness of this variety in northern climates. This consistent performance indicates the reliability of the ‘Frontenac’ grape cultivar in cold climate viticulture, and these results confirm the importance of this cultivar as documented in previous research (Kovaleski et al., 2018; Londo and Kovaleski, 2019). Contrastingly, as observed in our study, ‘La Crescent’s’ lesser cold hardiness, prompts a comparison with the work of Ferguson et al. (2014), who also reported lower cold hardiness in this cultivar. This observation highlights a possible vulnerability of the ‘La Crescent’ grape cultivar to cold stress and suggests the need for additional research on growing practices and breeding strategies to improve cold hardiness. The eight-year-old vines at a temperature of -9.4°C exhibited uniform cold hardiness, reflecting their cumulative hardiness developed over multiple seasons of frost and thaw. Within this context, the ‘Frontenac’ cultivar showed slightly better performance, particularly at the LT_50_ and LT_90_ thresholds. This slight but noticeable advantage in cold hardiness positions ‘Frontenac’ as a slightly more hardiness cultivar in the spectrum of survival under low temperature conditions. Yet, the statistical data (p-value of 0.946 for LT_10_, 0.549 for LT_50_, and 0.296 for LT_90_) sang of unity, with no significant differences to separate the cultivars in their collective stand against the cold (**Table 1**). This result indicated that parallels can be drawn with the research of Hemstad and Luby (2000), which suggested that certain cold hardiness traits might converge among mature vines under severe cold stress. The response of one-year-old vines, particularly the hardiness of ‘Frontenac’, highlighted the intrinsic varietal characteristics that confer cold hardiness from a young age. This result was in line with the work of Fennell (2004), who emphasized the genetic basis of cold hardiness and its expression even in younger vines. Our results indicated that at the extreme temperature of -17.8°C, the differences in cold hardiness among the grapevine cultivars became statistically insignificant, as evidenced by p-values of 0.814 for LT_10_, 0.747 for LT_50_, and 0.527 for LT_90_. This revealed that under severe cold stress, both one-year-old and eight-year-old vines of these cultivars exhibited a uniform level of hardiness to effectively withstand extreme cold conditions. In our study, the lack of significant differences among cultivars added an interesting dimension to the discourse on grapevine cold hardiness. This observation could be considered as an extension of the findings by Dami and Zhang (2023), who posited that extreme temperatures might elicit a uniform physiological response among different grape cultivars, leading to a homogenization of cold hardiness traits under such conditions. Also, our results highlighted the intricate interplay between environmental, genetic, and developmental factors in determining the cold hardiness of these grape cultivars, echoing the complex and multifaceted nature of this research area as noted by researchers such as Fennell (2004) and Beheshti et al. (2017). Our cold hardiness research, obtained through determination of LT values and statistical analysis, strengthened our understanding of the tolerance of these grape cultivars to variable autumn temperatures. This research has laid the foundation for future studies, and these findings, which highlight the diverse response of these grape cultivars in terms of hardiness and susceptibility, invite further research. They contribute to the evolving narrative of viticulture, challenging us to engage more deeply and carefully in ongoing research.

### Comparative assessment of lethal temperatures across grape cultivars based on vine age (eight-year-old and one-year-old) at different sampling temperatures

In our results, the lethal temperatures (LT_10_, LT_50_, and LT_90_) at which 10%, 50%, and 90% bud mortality occurred were crucial in unveiling the nuanced differences among these cultivars and vine ages (**Figures 4**–**6**). These results offer a significant and unique contribution to the field of viticulture research, with a specific focus on examining the complex nature of cold hardiness in the grapevine cultivars ‘Itasca’, ‘Frontenac’, ‘La Crescent’, and ‘Marquette’. We investigated the intricate relationship between vine age and cold hardiness, like a vintner delicately balancing the flavors in a fine wine. Our research journey into the cold hardiness of these cultivars uncovered a fundamental narrative: the increasing hardiness that comes with age. This exploration led to discoveries as detailed and multifaceted as the vines themselves. The eight-year-old vines demonstrated significantly enhanced cold hardiness compared to their one-year-old counterparts, indicating the concept that older contribute to increased hardiness against cold temperatures. This observation is consistent with previous studies showing the relationship between vine age and cold hardiness (Lisek, 2009; Lisek and Lisek, 2020; Antivilo et al., 2018). The ‘Frontenac’ cultivar showed notable cold hardiness, distinguishing itself as a particular hardiness cultivar in cold conditions. Its increased cold hardiness with age indicated a portrait of enduring hardiness, and this result was compatible with the results that have been said about vines throughout the ages and emphasized by some scientists (Seyedbagheri and Fallahi, 1995). Our research found that the ‘Itasca’, particularly in its older vines, displayed increased hardiness to cold temperatures, illustrating the beneficial impact of vine maturation on cold hardiness. Conversely, the ‘La Crescent’ and ‘Marquette’ showed a more variable response to cold stress, indicating a range of hardiness levels within these cultivars. This variability in cold hardiness among different cultivars is consistent with the results of authors who investigated the different responses of grapevines to cold conditions Hemstad and Luby (2000). While our study examined the cold tolerance of vines at -9.4°C, we observed that older vines exhibited more tolerance to cold conditions, repeating the findings of Seyedbagheri and Fallahi (1995). This trend became even more pronounced at the extreme temperature of -17.8°C, where the cold hardiness of older vines was significantly higher, and this result was consistent with findings of Fennell (2004). Our study not only advances the understanding of grapevine cold hardiness in viticulture science but also highlights the adaptive capacity of grapevines in the face of harsh winter conditions, contributing valuable insights to the field of cold climate viticulture.

### Comparison of the change in LT_50_ temperatures for buds in different grape cultivars at three sampling temperatures

In our extensive study of dormant bud cold hardiness, we determined LT_50_ values for ‘Itasca’, ‘Frontenac’, ‘La Crescent’ and ‘Marquette’ under various cold conditions. Our findings, particularly at -1.1°C, revealed significant intraspecific variability within the ‘Itasca’ cultivar. The variation in LT_50_ values of ‘Itasca’, ranging from -15.6°C in bud-3 to -21.8°C in bud-8, indicated the influence of both genetics and environmental conditions on cold hardiness. This is consistent with studies that point out similar varietal differences in the cold hardiness of this vine (Howell, 2001; Sanliang et al., 2002). The sensitivity observed in ‘Itasca’s’ buds-8 and buds-9 and the apparent hardiness in ‘Frontenac’s’ buds-1 and buds-7 further highlight the complexity of factors affecting cold hardiness in dormant buds. We observed profound changes in cold hardiness as temperatures dropped to -9.4°C, and ‘Frontenac’ and ‘La Crescent’ showed significant hardiness in buds-9. This variability, both within and between cultivars, is consistent with the findings reported by Wang et al. (2021). Our research extended into the extreme cold conditions of -17.8°C, revealing significant differences in cold hardiness among the grape cultivars (Kose et al., 2024). ‘La Crescent’ stood out, particularly in buds-8 and buds-9, showcasing an impressive hardiness that challenges the norm (Fennell, 2004). At the extreme temperature of -17.8°C, our study observed a substantial difference of -8.9°C in cold hardiness between the hardiest (‘La Crescent’) and the most sensitive (‘Itasca’), specifically in bud-8 (Buztepe et al., 2017). In juxtaposing our findings with the bud death rates across the same temperature spectrum, a consistent pattern of hardiness across all cultivars is evident, with only isolated instances of vulnerability (Bertamini et al., 2005). This hardiness, even at the harshest temperature of -17.8°C, indicates the robust nature of these cultivars, a characteristic that is crucial for viticulture in cold-prone regions (Bucur and Babes, 2016). On the other hand, our conclusion and assumption based upon the analysis of data presented in **Table 3**, is that an occurrence of 5% mortality in some buds is observed (Adsule et al., 2012). We hypothesize that this mortality rate is not attributed to cold-induced damage, but rather to instances of bud necrosis (Vasudevan et al., 1998).

### Comparison of LT_50_ temperature values according to the positions of the buds on the nodes of each grape cultivar

This study not only highlighted a spotlight on the influence of bud position on cold hardiness but also revealed the hardiness and vulnerabilities of ‘Itasca’, ‘Frontenac’, ‘La Crescent’, and ‘Marquette’ cultivars across different temperatures. The ‘Itasca’ at -1.1°C showed fascinating variability, with bud-8 emerging as the hardiest, a result consistent with previous findings showing that bud position plays an important role in cold hardiness (Kaya, 2020). The pronounced susceptibility of buds-3, followed by buds-1 and buds-6 in the ‘Itasca’ cultivar, introduced an additional layer of intricacy to our comprehension of its cold hardiness profile. When examining the more extreme temperature of 15°F (-9.4°C), the ‘Frontenac’ exhibited remarkable hardiness in bud-9, corroborating the observations of Howell (2001), which highlighted varietal disparities in cold hardiness. The reduced variability among other buds in ‘Frontenac’ may suggest a more uniform response to cold stress across this cultivar, a hypothesis that aligns with the findings of Mills et al. (2006). At the same temperature, the ‘La Crescent’ showed a striking display of hardiness in bud-9, which corroborates the findings of Bucur and Babes (2016), who emphasized the importance of cultivar selection in cold climate viticulture. The contrasting sensitivity of bud-4 at this temperature further shows the intricate interplay between bud position and cold hardiness. In the extreme temperature of -17.8°C, ‘Marquette’ presented a nuanced landscape of cold hardiness, with bud-6’s pronounced hardiness and bud-5’s sensitivity depicting a stark contrast within the same cultivar, a phenomenon that echoes the insights of Fennell (2004) into the complexities of grapevine cold acclimation. Our findings, when juxtaposed with previous studies, indicate the significant role of bud position in determining a grapevine’s cold hardiness, an observation that is particularly striking in the context of Bertamini et al. (2005), who delved into the adaptability of grapevines to cold environments. Our study introduces a novel dimension to this body of work by illustrating how different buds within the same cultivar respond uniquely to cold temperatures. In the realm of viticulture, it is a well-documented phenomenon that the lignification of buds in grapevines progresses acropetally, commencing from the basal bud. This developmental pattern, highlighted in the seminal work of Jones and Davis (2000), indicates the gradual hardening process in grapevine buds. Contrary to the established paradigm, our study unearthed intriguing findings that, in general, apical buds exhibited greater hardiness to cold compared to basal buds. This observation stands in stark contrast to prevailing theories in literature, which typically suggest that basal buds, having undergone earlier lignification, would demonstrate enhanced cold hardiness (Wolpert and Howell, 1985a). Moreover, as expounded by Keller (2020), posits that since hardening occurs later in upper buds, these should inherently show less endurance to cold stress. However, our findings compellingly diverge from this narrative, presenting an alternate reality where apical buds defy the expected trend of vulnerability. Our study delves into the realm of climatic fluctuations, a critical aspect often overlooked in grapevine cold hardiness research. Despite the assumption that pre-winter autumnal temperature variations could exacerbate cold damage, especially in more sensitive apical buds, our results paint a different picture. We demonstrated that in the studied cultivars, these climatic perturbations did not precipitate cold injury, a finding that resonates with the observations of Ferguson et al. (2014), who noted the hardiness of grapevines to varying pre-winter conditions. From an academic perspective, these findings lead to a re-evaluation of current assumptions regarding bud hardiness in grapevines and suggest that bud position is a more complex interaction between developmental timing and environmental factors than previously understood. This complexity not only adds a new dimension to our knowledge of grapevine physiology, but also adds practical meaning to viticultural practices. In this context, understanding the nuances of bud hardiness in different locations can help viticulturists make more informed decisions about pruning and protecting vines from cold damage.

### General evaluation

The utilization of PCA and correlation matrices offered profound insights into the complex interplay of cultivar characteristics and environmental factors in our exploratory journey through the landscape of grapevine cold hardiness. Our study’s innovative approach, employing these statistical tools, provided a nuanced understanding of cold hardiness across different grape cultivars and temperatures. The first two PCA biplots revealed intriguing patterns in the distribution of data points for different grape cultivars and sampling temperatures. In our cultivar centric PCA (**Figure 11A**), ‘Frontenac’ and ‘La Crescent’ presented more distinct profiles, reinforcing the notion of significant intraspecific variability in viticulture, as highlighted by Keller (2020).In contrast, the overlapping of ‘Itasca’ and ‘Marquette’ suggested a similarity in their variance, a finding that aligns with the observations of Clark et al. (2017) on the genetic and phenotypic similarities among certain grape cultivars. The PCA biplot for sampling times (**Figure 11B**) indicated a clear distinction between the data points at -17.8°C and those at the warmer temperatures of -1.1°C and -9.4°C. This is consistent with previous research on the effects of temperature on grapevine cold tolerance (Kaya and Kose, 2020). Our results from the correlation matrix (**Figure 11C**) indicated strong positive correlations between most of the buds, suggesting a consistent pattern of cold hardiness across different bud positions. This finding is particularly interesting as it differs from conventional wisdom and is consistent with previous research suggesting significant variability in cold hardiness depending on bud locations (Smith and Centinari, 2019). The absence of strong negative correlations further points to a uniformity in the cold hardiness response within each cultivar, a hypothesis that resonates with the work of Rubio et al. (2016). The heatmap (**Figure 11D**) indicated distinct patterns of bud LT_50_ values across different temperatures and cultivars, shedding light on the variability in cold hardiness both between and within the grape cultivars. The lighter shades for groups such as ‘Marquette’ at -9.4°C and ‘Itasca’ at -17.8°C, indicating higher LT_50_ values, contrasted starkly with the darker shades for ‘Frontenac’ at -1.1°C, representing lower LT_50_ values. This variability is consistent with the results of previous researchers highlighting different responses of grapevines to cold stress (Fennell, 2004).

## Conclusion

Our extensive investigation into the cold hardiness of grape cultivars such as ‘Itasca’, ‘Frontenac’, ‘La Crescent’, and ‘Marquette’ uncovered important insights into how these cultivars and their individual buds respond to changing autumn conditions and low temperatures. We observed distinct variations in cold hardiness not only between different cultivars but also among buds located on the same vine. Some buds of ‘Itasca’ and ‘Marquette’ exhibited greater resilience to extreme cold compared to others, indicating the significance of bud position in influencing the overall hardiness of a vine. Older vines generally performed better, especially at the critical LT_50_ and LT_90_ thresholds. In fact, the hardiness gap between younger and older vines was a major takeaway across all cultivars studied. The up-and-down autumn temperatures had a noticeable impact on the grapevines too. The swing between warm September days and chilly nights, followed by the drop in October/November, visibly influenced the cold hardiness limits we measured. Perhaps our most striking discovery upended traditional grape-growing doctrine: apical buds demonstrated superior cold-hardiness compared to basal buds. This pivotal discovery not only necessitated a reevaluation of long-standing viticultural practices, particularly pruning strategies, but also beckoned for an in-depth exploration into the physiological attributes that confer hardiness to these apical buds, potentially transforming grape cultivation in cold climates. Looking to the future, our study paves the way for further research in several areas. One potential avenue is the exploration of genetic or physiological factors that contribute to the observed differences in cold hardiness between cultivars and bud positions. Additionally, studies could focus on developing or improving viticultural practices that enhance the cold hardiness of grapevines, especially in the context of changing climate patterns and increasing occurrences of extreme weather events. This scholarly pursuit stands as a beacon, guiding us towards a deeper understanding and more resilient practice of viticulture in the face of climatic adversities.

## Data availability statement

The raw data supporting the conclusions of this article will be made available by the authors, without undue reservation. Requests should be made to the Corresponding Authors.

## Author contributions

OK: Writing – review & editing, Writing – original draft, Visualization, Validation, Supervision, Software, Resources, Project administration, Methodology, Investigation, Funding acquisition, Formal analysis, Data curation, Conceptualization. HD: Writing – review & editing, Software, Resources, Methodology, Investigation, Formal analysis, Data curation, Conceptualization. AS: Writing – review & editing, Software, Resources. CA: Writing – review & editing, Investigation, Formal analysis, Resources. HH-V: Writing – review & editing, Visualization, Validation, Supervision, Software, Resources, Project administration, Methodology, Investigation, Funding acquisition, Formal analysis, Data curation, Conceptualization.
